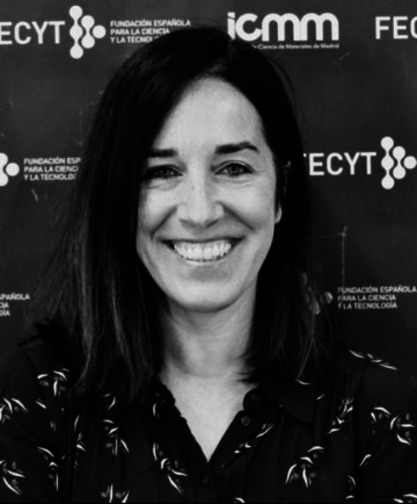# Understanding and Engineering Interfacial Adhesion in Solid‐State Batteries with Metallic Anodes

**DOI:** 10.1002/cssc.202202215

**Published:** 2023-04-19

**Authors:** Ieuan D. Seymour, Edouard Quérel, Rowena H. Brugge, Federico M. Pesci, Ainara Aguadero

**Affiliations:** ^1^ Department of Materials Imperial College London Exhibition Road SW7 2AZ London UK; ^2^ Instituto de Ciencia de Materiales de Madrid CSIC, Cantoblanco 28049 Madrid Spain

**Keywords:** alkali metals, electrode materials, first-principles calculations, interfaces, solid-state batteries

## Abstract

High performance alkali metal anode solid‐state batteries require solid/solid interfaces with fast ion transfer that are morphologically and chemically stable upon electrochemical cycling. Void formation at the alkali metal/solid‐state electrolyte interface during alkali metal stripping is responsible for constriction resistances and hotspots that can facilitate dendrite propagation and failure. Both externally applied pressures (35–400 MPa) and temperatures above the melting point of the alkali metal have been shown to improve the interfacial contact with the solid electrolyte, preventing the formation of voids. However, the extreme pressure and temperature conditions required can be difficult to meet for commercial solid‐state battery applications. In this review, we highlight the importance of interfacial adhesion or ‘wetting’ at alkali metal/solid electrolyte interfaces for achieving solid‐state batteries that can withstand high current densities without cell failure. The intrinsically poor adhesion at metal/ceramic interfaces poses fundamental limitations on many inorganics solid‐state electrolyte systems in the absence of applied pressure. Suppression of alkali metal voids can only be achieved for systems with high interfacial adhesion (i. e. ‘perfect wetting’) where the contact angle between the alkali metal and the solid‐state electrolyte surface goes to *θ*=0°. We identify key strategies to improve interfacial adhesion and suppress void formation including the adoption of interlayers, alloy anodes and 3D scaffolds. Computational modeling techniques have been invaluable for understanding the structure, stability and adhesion of solid‐state battery interfaces and we provide an overview of key techniques. Although focused on alkali metal solid‐state batteries, the fundamental understanding of interfacial adhesion discussed in this review has broader applications across the field of chemistry and material science from corrosion to biomaterials development.

## Graphical abstract

**Things can only get wetter**: Interfacial adhesion or ‘wetting’ between alkali metal anodes and solid‐state electrolytes (SSE) plays a central role in the performance of emerging solid‐state batteries. This Review provides a framework for understanding the fundamental interfacial chemistry that leads to adhesion at the alkali metal/SSE interface. Current strategies to control interfacial adhesion are reviewed, with a focus on how materials properties impact wettability.

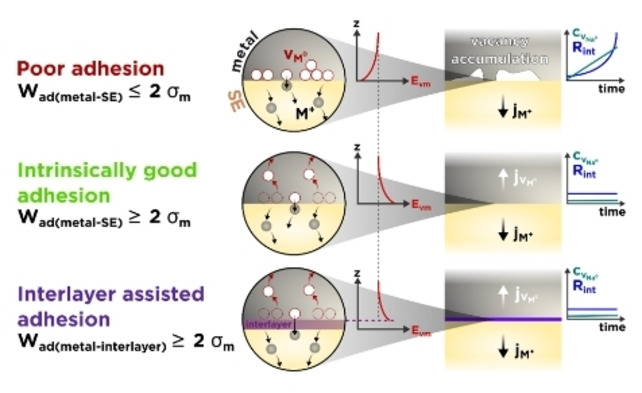

## 1 Introduction

Adhesion at the interface between two dissimilar materials is an eminently important subject that spans multiple research areas across the field of materials science and engineering. The fundamental concepts governing adhesion at heterogeneous interfaces apply to systems as varied as nanoparticle catalysis,[^1^, ^2^, ^3^, ^4^] semiconductors (metal/gate‐oxides),[^5^, ^6^] orthopedic implants, corrosion protection,^[7]^ and ceramic thermal barrier coatings for metallic turbine blades.[^8^, ^9^] In the field of Li‐ion batteries, the science of solid/solid interfacial adhesion has not been a key focus since heterogeneous materials are often held together by polymeric binders in cells employing liquid electrolytes. However, with research quickly progressing to new generations of solid‐state battery (SSB) cell architectures in which the electrodes and electrolyte are solids, solid/solid interfacial adhesion is becoming an increasingly important factor dictating the performance of batteries.

SSBs have attracted scientific and industrial attention because of possible improvements that they might provide in terms of cell energy density in comparison to Li‐ion batteries, particularly when alkali metals (Li, Na or K) are used as the negative electrodes.^[10]^ By virtue of the high specific capacity and low standard electrode potential of Li metal (3,860 mAh g^−1^ and −3.04 V), Li metal SSBs tend to be targeted for applications requiring the highest energy density (e. g., electric vehicles). Na metal has a lower specific capacity and standard electrode potential (1,160 mAh g^−1^ and −2.71 V), but a higher abundance and lower cost relative to Li. Thus, it is often predicted that Na metal SSBs will be primarily used for large‐scale stationary applications. With more and more reports suggesting that Na‐metal SSBs outperform Li metal SSBs in terms of power performance, the door is, however, still open for other applications beyond stationary storage.[^11^, ^12^] Solid‐state battery architectures have historically been assumed to be safer than liquid electrolytes containing a flammable liquid electrolyte. Recent studies have, however, suggested that solid‐state battery architectures, particularly containing high energy density Li metal anodes, may experience higher temperature rises during internal short circuit failures.^[13]^ Understanding and tailoring the interfacial chemistry to prevent failure through mechanisms such as dendrite growth (see Section 2.2) is therefore of upmost importance to maintain the safety of devices.

**Figure 1:**
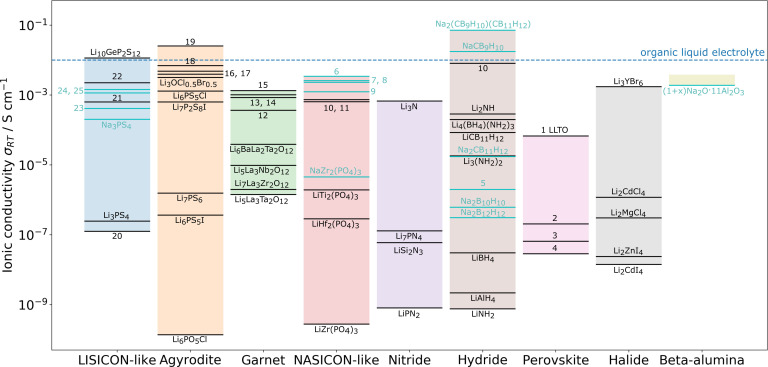
Examples of state‐of‐the‐art Li and Na SSEs and their conductivities. Key: 1. Li_0.34_La_0.51_TiO_2.94_; 2. LiSr_2_Ti_2_NbO_9_; 3. Li_0.06_La_0.66_Ti_0.93_Al_0.03_O_3_; 4. Li_0.34_Nd_0.55_TiO_3_; 5. Na_2_(BH_4_)_0.5_(NH_2_)_0.5_; 6. Na_3.1_Zr_1.95_Mg_0.05_Si_2_PO_12_; 7. Na_3_Hf_2_(SiO_4_)_2.2_(PO)_0.8_; 8. Na_3.1_Zr_1.9_Y_0.1_(SiO_4_)_2_(PO)_4_; 9. Na_3_Zr_2_(SiO_4_)_2_(PO)_4_; 10. Li_1.3_Al_0.3_Ti_3.7_(PO_4_)_3_; 11. Li_1.4_Al_0.4_(Ge_0.67_Ti_0.33_)_1.6_(PO_4_)_3_; 12. Li_7_La_3_Zr_2_O_12_ : 0.9 %Al; 13. Li_6.75_La_3_Zr_1.75_Nb_0.25_O_12_; 14. Li_6.4_La_3_Zr_1.4_Ta_0.6_O_12_; 15. Li_6.55_La_3_Zr_2_Ga_0.15_O_12_; 16. Li_7_P_3_S_11_; 17. Li_10_SnP_2_S_11_; 18. Li_6_PS_5_Br; 19. Li_9.5_Si_1.74_P_1.44_S_11.7_Cl; 20. Li_3.55_Si_0.5_P_0.5_O_4_; 21. Li_3.4_Si_0.4_P_0.6_S_4_; 22. Li_3.25_Ge_0.25_P_0.75_S_4_; 23. Na_10_SnP_2_S_12_; 24. Na_3_PSe_4_; 25. Na_11_Sn_2_PS_12_. Data has been taken from Refs. [14,17,20].

A wide variety of both Li and Na solid‐state electrolyte (SSE) chemistries have been studied to‐date, including inorganic electrolytes (oxides, sulfides, halides etc.; summarized in Figure 1) solid polymer electrolytes and polymer‐inorganic composite electrolytes. Through careful design of the structural chemistry, bulk ionic conductivities approaching or even exceeding those of organic liquid electrolytes (ca. 10^−2^ S cm^−1^) have been achieved in several sulfide‐based electrolytes, whereas state‐of‐the‐art oxide electrolytes such as the substituted garnet Li_7_La_3_Zr_2_O_12_ (LLZO) materials, have ionic conductivities around 10^−3^ S cm^−1^. There are many comprehensive reviews in the literature detailing and comparing the breadth of solid electrolyte classes, and the reader is referred to these for an in depth discussion.[^14^, ^15^, ^16^, ^17^, ^18^, ^19^]

**Figure 2:**
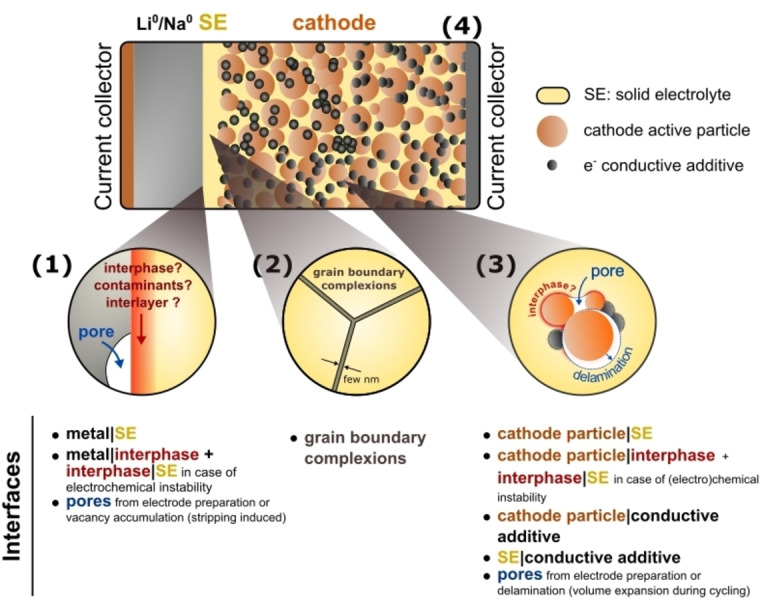
Summary of solid/solid interfaces in solid‐state batteries.

SSBs possess multiple solid/solid interfaces where adhesion is required. This includes both homogeneous interfaces (such as grain boundaries) and heterogenous interfaces (such as the metal/SSE interface). A nonexhaustive list of the types of interfaces present in metal‐SSBs and their specific adhesion challenges is provided below and schematically represented in Figure 2:

1. **Anode/SSE interface**: To enable efficient charge transfer, the interface between the alkali metal negative electrode and the SSE of a SSB needs to be in intimate contact over the entire lifetime of the cell. Careful control is required from the initial cell assembly step to ensure that a conformal interface is produced between the metal and SSE and that no ionically‐blocking contaminants are trapped during fabrication of the interface (see Section 4.2). Failure to produce a conformal interface will lead to a more tortuous path for charge transfer and consequently higher interface resistances. Due to their high chemical potentials, metallic anodes tend to reduce most SSEs. Upon reduction, a decomposition layer (interphase) typically forms at the metal/SSE interface (see Section 2.2.1). Although alkali metal anodes are the ultimate goal for SSB applications (Figure 2), conventional anode materials with lower capacities such as graphite or Si, or oxides such as TiNb_2_O_7_ or Li_4_Ti_5_O_12_ have also been considered. Solid/solid interfacial adhesion will also play an important role in these systems, particularly in the cases where large volume changes occur during electrochemical cycling, as is commonly observed in Si anodes for Li‐ion batteries.
2. **Grain boundaries**: Most SSEs are polycrystalline materials which are densified either thermally (sintering) or by applying pressure. In some cases, the intergranular region can have distinct chemical and structural properties across a thickness of a few nanometres in comparison to the adjacent grains.[^21^, ^22^, ^23^] These intergranular regions, sometimes called grain boundary ‘complexions’, arise from specific processing conditions. Controlling adhesion at the atomic scale can help to minimize the volume fraction of pores in SSEs and control the composition of complexions. This, in turn can have an important impact on the ionic and electronic transport properties of a SSE which, for instance, are thought to influence the ability to resist metal filament penetration.[^24^, ^25^, ^26^, ^27^]
3. **Cathode/SSE interface**: The positive electrode of a SSB is a composite structure consisting of redox active (cathode) particles, a fraction of SSE for ionic percolation and electronically conducting additives. The addition of polymeric binders may also be required if the electrode needs to be processed as a self‐standing film. Decomposition and the formation of interphases can occur at the interface between SSE and cathode particles if the two materials are not chemically or electrochemically stable with respect to each other. Pore formation can also occur between the cathode and SSE particles during processing or from delamination of the particles as a result of volume changes of the cathode during electrochemical cycling. The cathode/SSE interface is not further explored in this review; however, the reader is directed to other reviews on the topic.[^28^, ^29^, ^30^]
4. **Current collector**/**electrode interfaces**: These interfaces also need to be well bonded to ensure good electronic contact. Cu and Al foils are commonly used as the current collectors at the anode and the cathode of commercial Li‐ion batteries, respectively. As discussed in Section 2.1.4.1, the interfacial adhesion between metallic phases such as Cu and the Li or Na metal anode is expected to be strong, however the presence of impurities such as oxides layers at the interface between metallic phases can dramatically impact the interfacial properties. In metal SSBs assembled without a negative electrode (“anode‐free” cells), adhesion at the interface between the current collector and SSE needs to be considered. The nucleation and growth behavior of the anode during plating will depend on the binding energy between the anode metal and the current collector surface, which can be tuned by changing the composition of the target current collector material.^[31]^ On the positive electrode side, the current collector will be in contact with the various particles within the composite electrode.

These interfaces are all unique, both from their combination of materials and the way they are assembled (via sintering or mechanical pressing of materials) which makes the adhesion challenges slightly different in each case. The purpose of this review is to provide a scientific toolbox – including fundamental equations, computational modeling techniques and experimental strategies – to understand the nature of adhesion at interfaces in SSBs and design effective solutions. We focus more specifically on the alkali metal/SSE interface in this review, although the fundamental understanding of interfacial adhesion is more widely applicable to other interfacial challenges in battery materials.

In addition to the materials engineering challenge of creating intimate contact between the alkali metal and SSE during initial cell assembly, what makes adhesion at the metal/SSE even more complex to control is its dynamic instability during cycling (see Section 2.2.2). Upon discharge (stripping) of the alkali metal SSB, alkali metal atoms are ionized and hop to available crystallographic sites in the SSE, leaving behind a vacancy at the interface. Upon charge (plating) the reverse reaction occurs, and the alkali ion will be reduced at the interface to form a metallic atom. The coalescence of voids during stripping constricts the ionic current to increasingly smaller interfacial areas. This results in large overpotentials on stripping cycles and the risk of metal filament (dendrite) penetration during plating cycles (see Section 2.2.2.2).[^32^, ^33^] Preventing the coalescence of vacancies into interfacial voids during stripping has therefore become a central issue in SSB research.

To prevent the formation of interfacial voids, researchers have investigated the impact of changing external parameters, such as temperature or pressure, on cells (see Section 2.2.2.1). Although sometimes successful, these attempts at maintaining interfacial contact often require high pressure (from a few MPa to hundreds of MPa) or temperature (often close to the melting point of the metallic anode) to prevent void formation. The question of the transferability of these strategies to industrial battery cells, which often have thinner and more fragile layers, then becomes important to consider. For example, maintaining a 10 MPa pressure (roughly 100 × atmospheric pressure) on a 10 by 10 cm^2^ cell is a significant engineering challenge, as it would require an equivalent load of 10 T to be applied over this small area.

Alternatively, researchers can tailor the surface chemistry of the interfacing materials to improve their adhesion. Large‐scale simulations of static and dynamic interfaces are now a tool widely accessible to researchers thanks to the multiplication of supercomputers and the increase in their computing power. Modeling techniques to study interfaces are varied and have evolved rapidly in recent years (see Section 3). This review provides a summary of the fundamental aspects of these techniques, together with the most recent discoveries related to the adhesion of metal/SSE interfaces.

Controlling interfacial adhesion is one of the most important challenges limiting the power performance and longevity of SSBs which, to date, has been a significant hurdle to their commercialization. This review aims to provide researchers with the tools to engineer efficient solutions. The objectives of this review are:

- To provide a fundamental background to the concepts of wetting/adhesion at liquid/solid and solid/solid interfaces.
- To highlight the challenges associated with alkali metal/SSE adhesion in SSBs during electrochemical cycling and how this is impacted by interfacial structure and chemistry.
- To summarize the development of computational modeling techniques to simulate interfacial reactivity and adhesion and provide a review of state‐of‐the‐art computational results of interfacial properties of alkali metal/SSE interfaces.
- To highlight the most promising experimental solutions developed to improve adhesion at alkali metal/SSE interfaces.

## 2 The Theory of Wetting

### 2.1 What is wetting?

Interfacial adhesion describes the tendency of the surfaces of two dissimilar materials to stay together. The corresponding parameter, the work of adhesion *W*_ad_, is defined as the energy required to separate these two surfaces from one another. Analogously, the work of cohesion *W*_coh_, is defined as the energy to separate two surfaces of the same material. The term ‘wetting’ is often used interchangeably with interfacial adhesion in the literature. Historically, wetting has been used to describe the tendency for a liquid to stay in contact with a solid surface, but as will be discussed in the following sections, the concept of wetting and interfacial adhesion is readily extended to solid/solid interfaces between alkali metals and SSEs.

Interfacial adhesion combines an understanding of multiple electro‐chemo‐mechanical concepts from both the interfacial energy, γ , arising from the intrinsic properties of the materials making up the interface and any charge or chemical reorganization that occurs as the interface is created, and the mechanical strain originating from lattice misfit between the component phases.^[34]^ Therefore, focussing on the improvement of adhesion of interfaces by understanding the chemistry in addition to the microstructure and mechanical properties, is key to developing commercially viable solutions to the interface problem in all‐solid‐state batteries (ASSB).

#### 2.1.1 Surface energy

An important parameter for understanding wetting is the surface energy, σ , of a material. The surface energy of a solid material (S) is defined as the excess free energy required to create a surface of unit area in contact with vapor (V) by the process of breaking bonds (i. e. cleavage of the material). This surface energy, $\sigma_{SV},\;$ is given by Eq. (1), where *G* is the free energy of the system and *Ω* is the surface area, giving units of J m^−2^.

(1) $$\sigma_{SV}=\left(\frac{\delta G}{\delta \Omega }\right)$$

An analogous surface energy, $\sigma_{LV},$ for a liquid (L) in contact with its vapor can also be defined.

Because the process of creating a surface involves the breaking of bonds, it always requires work and is thus positive in value.^[35]^ The surface energy of a monoatomic crystal can also be thought of as the difference in potential energy of an atom at the surface ($E_{atom}^{surface}$ ) and a bulk atom ($E_{atom}^{bulk}$ ) divided by the surface area per atom *ω*, given in Eq. 2.

(2) $$\sigma_{SV}=\frac{E_{atom}^{surface}-\;E_{atom}^{bulk}}{\omega }$$

The magnitude of the surface energy depends strongly on the nature of the bonding in a material as bond cleavage is required in the surface creation process.

Values for the surface energies of different battery materials are shown in Table 1. Metals, such as Al, Ni and Cu, which form strong metallic bonds have high surface energies. Group 1, alkali metal, Li, Na and K form comparatively weaker metallic bonds due to the lower number of valence electrons available for bonding, resulting in low surface energies (0.472, 0.234 and 0.129 J m^−2^, respectively).[^35^, ^36^] The weaker metallic bonding in alkali metals is also seen through their characteristically low melting points (454, 371 and 336 K, respectively).[^35^, ^36^] The relationship between the surface energy of a metal and its melting temperature is well established.^[37]^

**Table 1:** Surface energies for common materials in solid‐state batteries.

System	Surface energy [J m^−2^]
Li^[a]^	0.472
Na^[a]^	0.234
K^[a]^	0.129
Al^[a]^	1.020
Ni^[a]^	2.080
Cu^[a]^	1.566
Li_2_O^[b]^	0.420
Na_2_O^[b]^	0.308
Al_2_O_3_^[b]^	0.606
*α*‐Al_2_O_3_^[c]^	2.64
*γ*‐Al_2_O_3_^[c]^	1.67

[a] Solid/vapor ($\sigma_{SV})$ surface energies for pure metals estimated from liquid/vapor surface $\sigma_{LV}$ energy at the melting temperature.^[38]^ [b] Surface tensions of molten oxides at the melting point.^[39]^ [c] Surface energies of Al_2_O_3_ polymorphs from calorimetry.^[40]^

**Figure 3:**
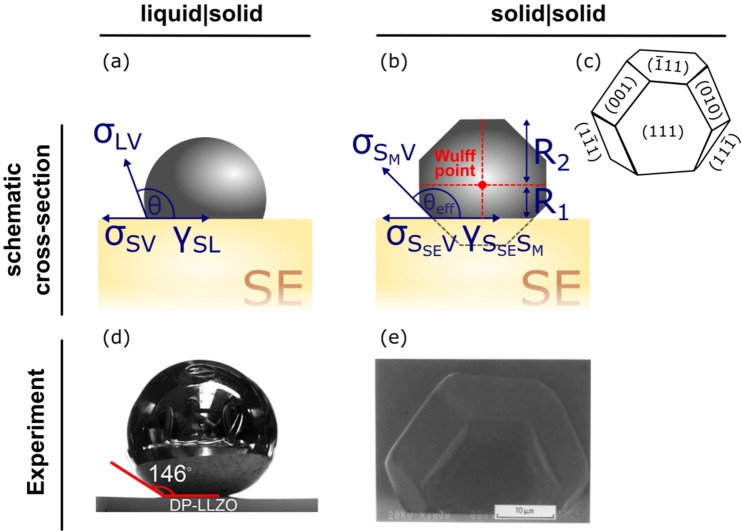
Types of interface classified by (a) solid/liquid and (b) solid/solid wetting, showing the Young's contact angle, θ , and the interfacial (*γ* ) and surface (σ ) energies. *R*_1_ and *R*_2_ are the distances from the Wulff point to the surface and the top facet, respectively. (c) An example of a single crystal equilibrated in contact with a flat solid substrate, resulting in an equilibrium Wulff shape with a central point given by the Wulff point. Adapted with permission from Ref. [41]. Copyright Springer Nature 2013. (d) Sessile drop example of a liquid on a flat horizontal surface. Reprinted with permission from Ref. [43]. Copyright 2017 American Chemical Society. (e) Example of the growth of a preferred orientation single crystal particle during annealing of solid copper particles on (0001) sapphire surfaces. Adapted with permission from Ref. [44]. Copyright 1988, Chapman and Hall Ltd.

The surface energy of a material also impacts the morphology of particles produced during synthesis or electrochemical deposition. Through knowledge a materials surface energy, the equilibrium particle shape can be predicted from a Wulff plot (Figure 3).^[36]^

#### 2.1.2 Wetting: Solid/liquid interfaces

Wetting is a term traditionally applicable to solid/liquid interfaces, and it describes the ability of a liquid to maintain contact with a solid surface, resulting from intermolecular interactions. The degree of wetting can be determined by the force balance between adhesive (amongst dissimilar particles) and cohesive (amongst similar particles) forces.

The wetting of a liquid on a solid surface is commonly assessed by a sessile drop experiment in which a hemispherical liquid droplet makes an angle of *θ* with the solid surface (Figure 3a).

Wetting can be described by the contact angle *θ* as follows: perfect wetting when *θ=*0° with ‘partial wetting’ anywhere between 0<*θ*<180°.^[41]^ In many previous studies, systems with 0≤ *θ*<90°, are described as having ‘good wetting’ with systems where 90<*θ* ≤ 180° described as having ‘bad wetting’.^[41]^ The latter nomenclature is, however, discouraged particularly as the use of the term ‘good wetting’ to describe contact angles anywhere up to 90° can be misleading when discussing the properties of alkali metal/SSE interfaces, as will be discussed in Section 3.2.4.2.

The Young equation [Eq. (3)] reflects the relative interfacial energies of a liquid/solid system under equilibrium (Figure 3a):

(3) $$\cos\theta =\frac{\sigma_{SV}-\gamma_{SL}}{\sigma_{LV}}$$

Additionally, the thermodynamic work of adhesion, *W*_ad_ is often used to compare the relative interfacial energies of a system. It is the work per unit area necessary to separate a solid/liquid interface with interfacial energy, $\gamma_{SL}$ , into two equilibrated surfaces of liquid‐vapor, $\sigma_{LV}$ , and solid‐vapor, $\sigma_{SV}$ [Eq. (4)]: 
(4)

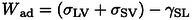




Combined with Young's equation, the work of adhesion can be expressed by the Young–Dupré relation (Thomas Young 1805, Anthanase Dupré and Paul Dupré 1869), as a function of the contact angle [Eq. 5]:^[42]^

(5)

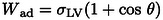




This is a useful form of the equation as, *θ* and $\sigma_{LV}$ are experimentally measurable values. However, it is also worth noting that most true solid surfaces are often macroscopically rough and chemically inhomogeneous, which can lead to scatter in contact angle data for the system, particularly when the liquid drop size is much larger than the surface defects on the substrate. A discussion of the sessile drop experiment methodology for obtaining contact angles is given in Section 2.1.6 of this review.

#### 2.1.3 Wetting: solid/solid interfaces

In the case of solid/solid interfaces, sessile drop experiments to measure the Young's contact angle are not comprehensive – the effect of the anisotropic crystal shape on the apparent contact angle is overlooked, despite playing a crucial role in the minimization of interface and surface energy (Figure 3b). The equilibrium crystal shape (also known as the Wulff shape)^[45]^ describes the orientation‐dependence of the surface energy of a crystal (Figure 3b and 3c). Winterbottom analysis^[46]^ can be used to measure experimentally the solid/solid interfacial energies for a crystal on a solid substrate as discussed further in Section 2.1.7. However, for such systems to be accurately measured experimentally, a single crystal small enough to be equilibrated on a region of substrate free of defects such as grain boundaries must be chosen, making measurement challenging.

The work of adhesion, *W*_ad_, for a solid/solid interface between two solid materials, *X* and *Y*, with interface energy $\gamma_{XY}\;$ can be defined in an analogous way to Eq. (4) [Eq. 6]:
(6)

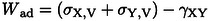




Where $\sigma_{X,V}$ and $\sigma_{Y,V}$ are the surface energies of phases *X* and *Y* in equilibrium with the vapor. For solid/solid interfaces, the work of separation, *W*_sep_, is also an important term and is used to define the difference in energy between an equilibrated interface and the two surfaces created immediately after the interface has been separated (i. e., before they have reached equilibrium).^[41]^ Since the surface energy is at a minimum at equilibrium, the work of separation is larger than the work of adhesion.

**Figure 4:**
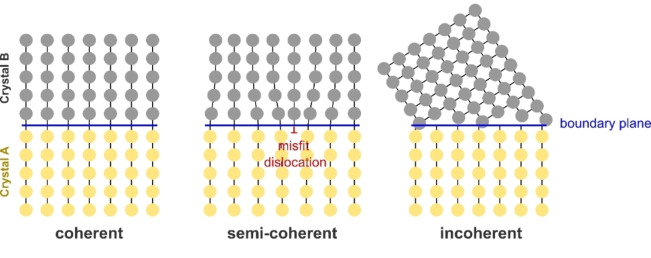
Schematic depiction of 3 possible types of interfaces between two crystalline solids: coherent, semicoherent, and incoherent. Atoms in the two phases are shown in yellow and grey. Boundary plane between the two phases is shown as a blue line and the dislocation line for the semicoherent interface is shown in red.

For crystalline solids with long‐range periodic structures, the relative atomic arrangements between atoms at solid/solid interfaces have an important influence on the interfacial structure and energetics. The interface between two solid materials *X* and *Y* is typically classified into one of three types: coherent, semicoherent or incoherent (Figure 4). If phases *X* and *Y* are cubic and have lattice parameters *a*_X_ and *a*_Y_, the degree of misfit, δ, is defined as:^[47]^ $\delta =\frac{2(a_{X}-a_{Y})}{\left(a_{X}+a_{Y}\right)}$ . A coherent interface occurs when the degree of misfit is small (δ <5 %) and the atomistic configuration of the two phases are the same across the interfacial plane, which typically only occurs for specific surface orientations, for example between analogous planes of face centred cubic (FCC) Al_3_Li precipitates and Al metal.^[48]^ If the misfit is small between the bulk lattice parameters of *X* and *Y*, once they are brought into contact at the interface, a strain may develop in one or both of the phases to maintain coherency, such as in the LiFePO_4_/FePO_4_ cathode system on delithiation.^[49]^ When the misfit strain between the two phases is larger, a semicoherent interface can be formed in which periodically spaced misfit dislocations are introduced to accommodate the lattice mismatch, which separate regions of near coherent interface.^[47]^ An incoherent interface occurs when the lattice parameters and/or atomistic structure of the interface differ significantly, which results in a lack of matching at the interface boundary. As recently outlined in Ref. [50], the majority of Li SSE materials are expected to form incoherent interfaces with body centered cubic (BCC) lithium metal.

Importantly, the atomic arrangement of the interface has a big impact on the work of adhesion (*W*_ad_). At a coherent interface, the atoms within the interfacial plane experience a uniform bonding environment on either side of the interface. In a semicoherent or incoherent interface, there are a distribution of different bond lengths between interfacial atoms. The large lattice mismatch in an incoherent interface or introduction of misfit dislocations in a semicoherent interface typically weakens and reduces the density of interfacial bonds relative to an analogous coherent interface.^[51]^ Coherent interfaces therefore intrinsically have higher *W*_ad_. The inhomogeneous nature of the bonding environments in incoherent interfaces has also been suggested to impact the degree of covalency/ionicity of interfacial bonds in metal/ceramic systems.^[52]^ Computational modeling has been particularly useful for analyzing the variation in the interfacial adhesion as a function of the degree lattice mismatch, as discussed further in Section 3.2.3: ‘Explicit interface calculations’.

#### 2.1.4 Reactive vs nonreactive wetting

The wetting behavior between two phases can be broadly classified into one of two regimes: reactive wetting or nonreactive wetting.^[35]^ Reactive wetting can occur when there is a thermodynamic driving force, i. e. a negative Gibb's free energy Δ*G*, for components *A* and *B* to react to form a new phase, for example *A*+*B*→*AB*. For instance, the formation of interphases in SSBs in contact with alkali metals (see Section 2.2.1) can be a source of reactive wetting.

In contrast, nonreactive wetting occurs when there is no thermodynamic driving force for components A and B to react to form a new phase (positive ΔG ), although an interaction is still present at the interface between the components which leads to adhesion (in the case of metal/metal interfaces, this could be a metallic bond). For nonreactive wetting, the strength of the adhesion between two components, as measured by the contact angle *θ* in Eq. (3), is sensitive to the electronic structure of the materials. The Young–Dupré equation in Eq. (5) is in fact only strictly applicable for nonreactive wetting, in the sense that it applies only to a nondynamic interface in equilibrium.

In sessile drop tests of liquid/solid systems, the distinction between reactive and nonreactive wetting is often made based on the time‐evolution of a droplet of radius *R*(*t*) and contact angle *θ*(*t*). In a nonreactive couple, using the assumption that spreading depends on the competition between capillary forces driving, and liquid viscosity attenuating the wetting process, the spreading rate follows Tanner's law, *R*(*t*)≈*t* ^1/10^ and *θ*(*t*)≈*t* ^−3/10^.^[53]^ This relationship leads to very rapid equilibration on the order of 10^−1^ s for the contact angle of nonreactive liquid metals on solid surfaces for contact angles exceeding 20°.^[54]^ Longer equilibration times are found when the contact angle gets closer to zero.

In couples far from chemical equilibrium, reactive wetting results in interfacial reactions leading to phase formation, which can strongly modify the chemistry, structure, and topography of the interface. These changes in turn affect the degree of wetting and thus can be exploited in practice to control wetting and adhesion. Strategies to improve wetting through the introduction of reactive interlayers are described in detail in Section 4. Because droplet dynamics are different in reactive wetting systems, the spreading process is often characterized by a linear dependence of the droplet radius with time, *R*(*t*)≈*t*.[^55^, ^56^]

The effect of temperature on spreading kinetics can be pronounced in reactive wetting systems. A change in temperature can affect surface adhesion, reaction rate and viscosity of the liquid phase.^[57]^ The temperature‐dependence of the reaction rate (Δ*G* for reaction) is likely to be the largest manifestation of the influence of temperature in a reactive wetting system. In metal/metal systems where the excess entropy at the interface may be significant, or in systems with high reactivity, higher temperatures can significantly speed up the process of reactive wetting and the entropy term can contribute significantly to the overall interface energy.

In the following section, an overview of the atomistic mechanisms that govern adhesion at metal/metal and metal/nonmetal interfaces for both reactive and nonreactive systems is given.

##### 2.1.4.1 Metal/metal interfaces

For a system of two dissimilar metals, *A* and *B* with limited solubility, even in the absence of a thermodynamic driving force to form a new compound *A*_x_ *B*_y_, the strong metallic bonding between metals leads to a strong adhesion and thus a small *θ*.^[58]^ For example, the Cu/W couple has a contact angle as low as 10° under reducing atmospheres and a very limited solubility of W in molten Cu (a few ppm).^[35]^ Al‐Na is another example of a nonreactive metal‐metal system which is relevant to battery materials, in which minimal dissolution of Al in Na occurs.[^59^, ^60^]

Typical values of interfacial energy in metal/metal couples are in the range 0.05 to 0.5 J m^−2^, and the work of adhesion *W*_ad_, is ∼1–5 J m^−2^. In these systems, nonreactive interfaces form quickly (<100 ms timescales for spreading of millimetre sized droplets).^[35]^ In the case of reactive wetting at liquid metal/metal interfaces, where dissolution is generally controlled by diffusion in the liquid alloy, a stable configuration is reached much more slowly (ca. 1000 s) for millimetre sized droplets. The contact angles are usually slightly lower in reactive systems with mutual solubility compared to nonreactive systems.

Wetting may sometimes be improved by the addition of elements to the metal melt. For example, in Li metal batteries, Ag doping into Li to form a low atomic % doped alloy was shown to have reduced chemical reactivity and improved dry air stability,^[61]^ whilst adding 30–40 % Zn to Cu appreciably alters the contact angle behavior with Ag.^[60]^ In a liquid metal/solid metal couple, if the surface energies of the alloying elements in the liquid are much lower than that of the pure liquid metal, a net decrease in the surface energy can occur for the resulting liquid metal alloy. According to the Young equation [Eq. (3)], this decrease in *σ*_LV_ would reduce the contact angle, *θ*, towards the perfect wetting situation on the solid if the solid surface energy and interfacial energy, *σ*_SV_ and *γ*_SL_, respectively, are constant. This is seldom seen experimentally, implying that in these cases, the adsorption of certain alloying elements leads to both a decrease in *σ*_LV_ for the liquid but also in *σ*_SV_ for the solid, with the resulting effect on the contact angle being negligible. This highlights that an increase in wetting is seldom produced by additives with low‐melting points and low surface‐energies.^[35]^

Note that metals are very sensitive to impurities, especially oxygen, and this can strongly affect the surface properties. When a layer is formed on the surface, a nonwetting contact angle may be observed. The sensitivity to the environment (i. e. oxygen partial pressure) is relevant for alkali metal anode based batteries during the preparation and handling of the metal.^[43]^ The strong adsorption of oxygen on metal surfaces can decrease *σ*_SV_ significantly, but this effect is often anisotropic. Reductions in *σ*_SV_ have also been observed with additions of very small amounts of low‐surface‐energy metals such as Sb or Bi in Cu.^[35]^ Adsorption at the solid surface is driven by the relaxation of the lattice strain energy when using solutes with larger atomic sizes than that of the solvent. For example, in the Al/WC interface, the introduction of Li and Mg as alloying agents lead to a reduction in *W*_ad_ by 1.5–2.5 J m^−2^, as a result of strain effects caused by their large size; however, Li impurities caused a greater reduction than Mg despite its smaller size, indicating the importance of the role of electronic defects.^[62]^ Often this contribution to the adsorption energy is more important compared to other contributions such as the cohesion energy of the pure metal and chemical *A*–*B* interactions.

##### 2.1.4.2 Metal/nonmetal interfaces

The theory of bonding between metal and ceramic materials has been developed for over half a century due to the importance of metal‐ceramic interfaces in a diverse range of engineering and scientific fields. These range from microelectronics and magnetoresistance devices,^[63]^ to thermal barrier coatings or corrosion protection layers for jet engines and power generation,^[9]^ to sensor technology and nanocatalysts,^[64]^ medical implants and dentistry, through to structural materials for nuclear reactors.^[65]^ Analogous to the metal/metal interfaces described previously, metal/ceramic interfaces can be grouped into reactive and nonreactive systems based on whether there is a driving force for a ceramic *A*_x_O_y_ to dissolve in the liquid or solid metal *M*, or form a secondary phase via a reaction such as *A*O_y_+*M*→*M*O_y_+*A*.

Although the majority of previous studies on the fundamental principles of adhesion at metal/ceramic interfaces have focused on the interactions at metal/metal oxide systems, there has also been significant work on metal/non‐oxide interfaces, including carbides, nitrides and halides, which are relevant to the diverse range of material chemistries studied in ASSBs.[^66^, ^67^, ^68^, ^69^, ^70^] The nature of bonding in different families of ceramic materials vary considerably, with primarily ionic bonding in wide bandgap oxide and halide materials such as Al_2_O_3_ and LiF, to covalent‐type bonding in materials such as SiO_2_,^[8]^ to metallic bonding in materials such as transition metal nitrides, borides, carbides and WC.^[62]^ In semiconductors such as Si, Ge or SiC, free surfaces also display a metallic character.^[54]^

**Figure 5:**
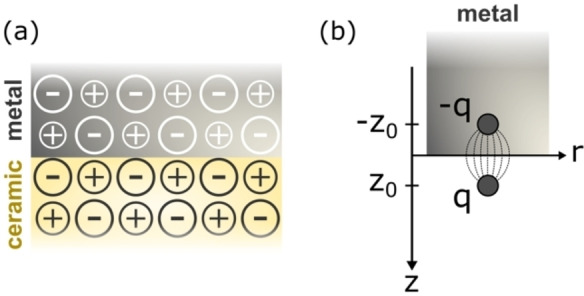
(a) Schematic depiction of ceramic/metal interface in which the nonpolar ceramic is a lattice of positive and negative charges interacting with a conductive surface of a metal, with an image plane at position *z*_0_. Adapted with permission from Ref. [72]. Copyright 1992 Elsevier. (b) Interaction between a point charge (*q*) and a metal surface.

An early theoretical approach that was developed to understand the interaction between the surface of nonreactive metals and wide band gap ionic metal oxide materials was the image interaction model.[^1^, ^71^, ^72^, ^73^] In this model, the ceramic is treated as a lattice made up of positive and negative charges that interact with a conductive surface of a metal (Figure 5a). From classical electrostatics, a point charge *q* at a distance *z* from a metal surface is attracted by its own image by an interaction energy given by Eq. 7:
(7)

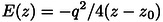




where *z*_0_ is the position in the image plane (coinciding with the geometrical surface of a classical conductor, Figure 5b).^[1]^

The success of the image interaction model is that it predicts values for the work of adhesion that are of the order of J m^−2^, which are typical of experimentally‐observed values for metal/oxide interfaces, suggesting that electrostatics play an important and often dominant role in the adhesion between metal and (nonpolar) oxide surfaces.^[1]^ The dominance of physical, electrostatic forces at the metal‐oxide interface over covalent interactions was also supported in an early study by Eustathopoulos and Drevet in which they showed that the contact angle for different nonreactive metal/Al_2_O_3_ interfaces correlated with the liquid/vapor surface energy of the metal *M*, but not with the enthalpy of formation of possible metal oxide *M*O_y_ phases formed at the boundary.^[74]^ However, for a number of other metal‐ceramic interfaces, it has been argued that short range covalent or metallic bonding effects can dominate the adhesion. Over the last three decades, ab initio calculations based on density functional theory have allowed a more detailed understanding of the nature of the local electronic structure and adhesion at metal ceramic interfaces, as will be discussed further in Section 3.

At the interface of Ni with Al_2_O_3_ and SiO_2_, it was suggested from first principles calculations by Jarvis and Carter,[^8^, ^9^] that the more covalent SiO_2_ leads to a decrease in the Ni 3*d* shell repulsions with O, leading to three times stronger adhesion than with the more ionic Al_2_O_3_. Both SiO_2_ and Al_2_O_3_ oxides are often the subject of fundamental studies as they can be obtained easily as high purity solids. In another first principles study, it was shown that the presence of metals with open *d*‐shells such as Zr or Ti enhance the adhesion at the interface, both in the metal phase (e. g., Zr‐Al_2_O_3_) and in the metal oxide (i. e. Ni‐ZrO_2_).^[9]^ The increased adhesion introduced by open *d*‐shell elements was due to the ability for the elements to accept electrons across the interface, which reduced closed‐shell repulsions and facilitated bonding with oxygen anions.

The mole fraction of oxygen, X_O_, in the metal has been used to classify metal‐oxide systems as nonreactive or reactive by Eustathopoulos and coworkers.^[35]^ For values of X_O_ <10^−6^, systems can be considered nonreactive in terms of wettability, and have weak physical metal/oxide interactions at a locally sharp interface (examples include Cu, Ag, Au and Ga on SiO_2_ and Al_2_O_3_). For X_O_ >10^−5^, the contact angle decreases and tends to zero as reactivity increases. The type of reaction includes dissolution reactions (such as with Cu/NiO) and reactions which form new phases at the interface (such as Ti/MgO and Zr/MgO). The adsorption layers (<1 nm thick) formed in the first case can provide significant improvements to wetting.

In the case of reactive wetting in metal/nonmetal interfaces by thermodynamically favorable secondary phase formation, Landry and Eustathopoulos^[55]^ proposed a model to describe the formation of continuous layers of a new compound.^[54]^ According to their model, the final contact angle, *θ* ^1^, given by Eq. (8), is governed by the Gibbs free energy for the reaction, Δ*G* and the change in the interfacial energy Δ$\gamma_{SL}$ , where *θ* ^0^ is the equilibrium contact angle in the absence of reaction:^[75]^

(8) $${\cos\theta }^{1}={\cos\theta }^{0}-\frac{{\Delta \gamma }_{SL}}{\sigma_{LV}}-\frac{\Delta G}{\sigma_{LV}}$$

Strategies utilizing reactive wetting as seen in the SSB literature are discussed in detail in Section 4: for example, the Al_2_O_3_ interlayer adopted by Han et al.^[76]^ at the Li/Li_7_La_3_Zr_2_O_12_ interface is an example of reactive wetting between Li and Al_2_O_3_. In other fields, the wetting behavior of Ni−Si alloys on vitreous carbon was shown to vary with Si content as a result of reactive wetting to form SiC which occurs above 35 at% Si at 1200 °C and which subsequently leads to improved wetting and a reduction in the contact angle.[^54^, ^77^]

It is worth mentioning that native oxide films and reactive layers found on many ceramic surfaces following certain environmental exposure conditions act as wetting barriers, leading to nonwetting with nonreactive liquid metals (e. g., Li_2_CO_3_ on Li_7_La_3_Zr_2_O_12_ after exposure to CO_2_ in air, see Section 4.2 for further details).[^78^, ^79^, ^80^, ^81^, ^82^, ^83^] Correspondingly, reactivity can also lead to the formation of a reaction product less wetted than the initial substrate, i. e. to the formation of a wetting barrier.^[54]^ In the brazing field, the Au‐TiC system provides examples of this type of wetting barrier behavior.[^54^, ^84^] Here, pure Au does not wet TiC (*θ*=130°, the same value as that for Au on carbon). Due to the strong interaction between Au and Ti, slight dissolution of Ti from the substrate into Au occurs. As the solubility of C in Au is much lower than Ti, graphite precipitates at the interface and acts as a wetting barrier. However, the addition of a small amount of Ni can increase the solubility of C (the solubility of C in Ni is much greater than in Au), preventing the formation of the graphite layer on TiC. For doping levels of 3–7 at.% Ni, the contact angle decreases to 60–80°; Ni here removes the wetting barrier through dissolution.

Finally, the concept of electrowetting, that is, altering the interface adhesion by an applied potential, may be used to cause a change in the wetting properties, by creating a decrease in the interfacial energy of the metal/electrolyte interface due to the presence of excess charges on the metal surface.[^85^, ^86^, ^87^]

#### 2.1.5 Influence of morphology and chemical heterogeneity on wetting

As described in the previous section, whilst Young's equation is based on rigorous theoretical concepts, its applicability to actual experiments is extremely limited. The conditions of a smooth, homogeneous surface are rarely met experimentally. Nature provides us with numerous superhydrophobic/philic surfaces in which morphology results in deviations in Young's equation. The two most famous examples are the lotus leaf and rose petal effects: the lotus leaf effect characterizes surfaces whose micro/nano hierarchical morphology is such that trapped air pockets will make liquids appear to “float” on top of them; such hierarchical structures can also result in the opposite effect (the rose petal effect) when liquids penetrate and impregnate the microstructural pores.^[88]^

A first modification to Young's equation was introduced by Wenzel^[89]^ to account for the roughness of solid substrates. Let *R*_f_ be the ratio of the total surface area of a solid to its flat projected area (*R*_f_ >1), the apparent contact angle defined by Wenzel (*θ*_W_) is related to the Young's contact angle (*θ*_Y_) by Eq. 9:

(9) $$cos\theta_{W}=R_{f}cos\theta_{Y}$$

Later, Cassie and Baxter modified Young's equation to include cases where air pockets are trapped under the droplet [Eq. (10)]. When air pockets are present, the solid has a fraction *f*_1_ of its surface in contact with liquid and a fraction *f*_2_ in contact with air (with *f*_1_+*f*_2_=1). The apparent contact angle defined by Cassie and Baxter (*θ*_CB_) is related to the Young's contact angle by:

(10) $$cos\theta_{CB}=f_{1}cos\theta_{Y}-f_{2}$$

**Figure 6:**
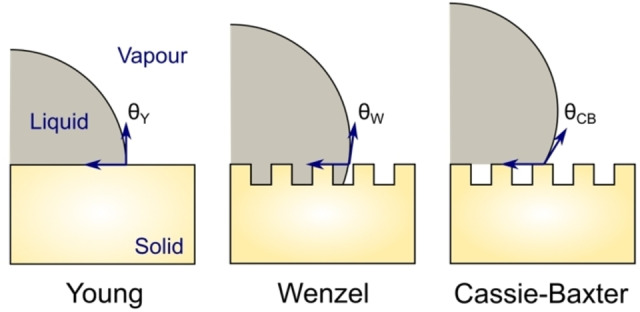
Schematic representation of liquid droplet contact angles (θ ) described by the Young (Y), Wenzel (W) and Cassie–Baxter (CB) equations.

The contact angles resulting from the Wenzel and Cassie–Baxter equations are shown schematically in Figure 6. When there are no air pockets on the surface, *f*_2_ is zero and Eq. (10) becomes identical to Wenzel's equation with *f*_1_=*R*_f_. The short derivation for Eq. (10) can be found in Cassie and Baxter's original publication.^[90]^

#### 2.1.6 Contact angle measurements at liquid metal/SSE interfaces

Contact angle (CA) measurements are extremely useful methods to extract quantifiable information about wetting using low‐cost instruments and simple procedures. Unsurprisingly, the SSB community has made efforts to adapt CA measurements for the characterization of wetting at alkali metal/SSE interfaces. This is usually achieved by melting alkali metals and using conventional CA measurements methods for liquid/solid interfaces. Contact angles can be measured via numerous methods including the sessile drop, the tilting plate or immersion‐emersion methods.^[91]^ To our knowledge, publications reporting CA measurements for alkali metal/SSE interfaces have all employed the sessile drop method. Whilst CA measurements can be a source of quantitative information, they are often perceived in the field of SSBs as unreliable due to their poor repeatability and reproducibility. Therefore, CA measurements are most often used only to provide qualitative information. The poor reproducibility of CA measurement experiments is caused by multiple factors whose detrimental role can be mitigated by careful control of measurement conditions.

**Figure 7:**
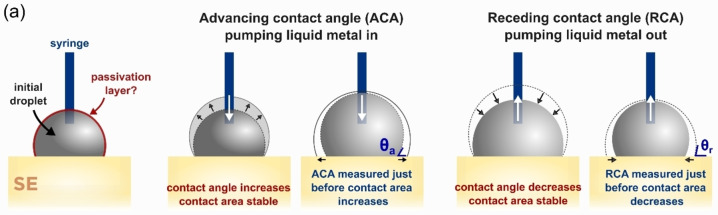
How to measure advancing and receding contact angles at a liquid/solid interface.

The first misconception to deconstruct is the idea of an “intrinsic” or “equilibrium” contact angle, as defined in the Young–Dupré equation. The Young contact angle *θ*_Y_ applies to nonreactive liquids spreading on a flat, perfectly smooth, and chemically homogenous solid substrate. Experimentally, such surfaces are extremely difficult to obtain. Contact angles always display a hysteresis resulting from the roughness and surface chemical inhomogeneities of the solid substrate. Hysteresis is defined as the difference between the advancing and the receding contact angles (*θ*_a_–*θ*_r_).^[92]^ The advancing and receding contact angles define the range of metastable contact angles which a liquid can adopt on a solid (Figure 7). Experimentally, the advancing contact angle is defined as the maximum angle that a droplet adopts on a solid before spreading (i. e. before the droplet's diameter increases to reach a new metastable position), while the receding contact angle is the minimum angle that the droplet adopts before retreating. Using the sessile drop method, these angles can be measured by pumping liquid in or out of the droplet using a syringe and simultaneously filming the changes in the droplet's shape. The issue of contact angle hysteresis is well documented in other fields of research where wetting is studied.[^91^, ^92^, ^93^]

Advanced setups such as the one adopted by Liu et al. for their study on the wetting properties of Galinstan (a fast oxidizing liquid metal alloy) should be considered to give quantitative analysis of wetting in these interfaces.^[86]^ The sessile drop CA measurement equipment was fully enclosed in a glovebox with controlled atmosphere. The use of a syringe driver allowed dynamic CA measurements to be acquired and advancing/receding contact angles to be obtained. In the case of alkali metals, which are solid at room temperature, the setup would have to be modified to include a heated metal syringe^[43]^ or capillary^[94]^ to deposit a droplet in a controlled manner. Because variations of even a few ppm can have a significant effect on the wetting properties of easily oxidizable liquid metals,[^95^, ^96^] the O_2_ concentration in the glovebox should always be reported.

#### 2.1.7 Measuring solid/solid interface energies: Winterbottom analysis

In the following section, an introduction to the measurement of interfacial energy between two solids through Winterbottom analysis^[46]^ is given, with a focus on metal/ceramic interfaces. For a more in‐depth discussion, the reader is directed to the work by Kaplan et al.^[41]^ The eponymous “Winterbottom analysis” relies on a geometrical analysis of the Wulff shape (Figure 3b) of a single crystal equilibrated on a solid substrate under conditions of constant temperature, volume and chemical potential.^[46]^ Only two characteristic distances need to be measured to determine the interface energy of a single crystal equilibrated on a solid substrate. These two characteristic distances, which are represented in Figure 3b, are: the distance between the crystal Wulff point and the interface with the substrate (*R*_1_), and the distance between the Wulff point and the top facet of the crystal (*R*_2_). The substrate/particle interface energy ($\gamma_{SP}$ ) can then be calculated via Eq. (11), provided that the surface energies of the substrate ($\sigma_{SV}$ ) and of the facet of the particle in contact with the interface ($\sigma_{PV}$ ) are known.

(11) $$\frac{R_{1}}{R_{2}}=\frac{\gamma_{SP}-\sigma_{SV}}{\sigma_{PV}}$$

Importantly, Eq. (11) is only valid for single crystal particles, whose interface with their substrate is perfectly flat and coplanar with the substrate surface (meaning the particle should rest on the surface and not be socketed in the substrate). Unfortunately, the technique can also only be used to calculate interface energies in systems with weak adhesion, because the Wulff point of the equilibrated particle should be above the interface for it to be accurately identified (i. e., effective contact angle contact angle larger than 90°, or $\gamma_{SP}-\sigma_{SV}>0$ ).

Although Eq. (11) is quite simple, the associated experimental protocol to access and measure the two characteristic distances *R*_1_ and *R*_2_ is rather complex. A detailed methodology was developed by Kaplan et al. and applied to determine the interface energy of Au and Ni single crystals equilibrated respectively on sapphire and yttrium stabilized zirconia (YSZ).[^97^, ^98^] The main steps of their protocol are summarized below.

In both cases, Au and Ni were first sputtered as a thin layer to cover their respective substrates (sapphire or YSZ). The samples were then heated slightly above the melting point of the metal to dewet the sputtered film and form liquid droplets on the substrate surface. Faceted solid particles were obtained from these liquid droplets upon cooling via a controlled temperature profile. The orientation of the particles on their respective substrates can be studied using XRD and/or electron backscatter diffraction (EBSD).^[98]^ To study the specific interface orientation relationship, small particles were selected for TEM specimen preparation by focused ion beam (FIB) milling. The particles were cross‐sectioned in such a way that the substrate is positioned in a low‐index zone axis for the TEM analysis. After orienting the TEM specimen with the interface parallel to the incident electron beam, selected area electron diffraction (SAED) patterns were collected to determine the interface orientation relationship. The interface energy for this specific interface orientation can then be calculated by measuring the distances R_1_ and R_2_ from the TEM micrographs.

To our knowledge, this methodology has not yet been applied to study alkali metal/solid electrolyte interfaces. Alkali metals are very reactive and tend to rapidly form a passivation layer on their surface even under ultra‐high vacuum conditions; the steps of sputtering, dewetting and FIB sectioning would therefore have to be carried out quickly and in ultra‐high vacuum conditions after which, the surface would need to be coated with a protecting layer. The presence of a passivation layer could create a resistance to dewetting and/or lead to the equilibration of crystal Wulff shapes with non‐lowest energy. In addition, vaporization of the alkali metal during the dewetting step should be prevented. If these challenges can be overcome through experimental method development, Winterbottom analysis may also be a powerful tool for studying the adhesion at alkali metal/solid electrolyte interfaces.

### 2.2 The role of interfaces and wetting in metal anode solid state batteries

#### 2.2.1 Thermodynamics of the SSE/metal anode interface

**Figure 8:**
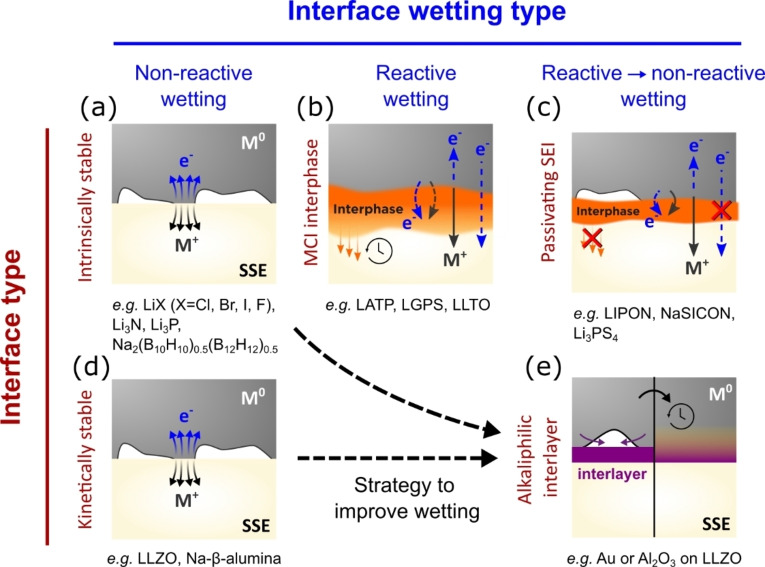
Types of alkali metal/SSE interface in an SSB and interphase formation: (a) Thermodynamically stable interface (no instantaneous chemical decomposition). (b, c) Thermodynamically unstable interfaces, where (b) shows SSE decomposing on contact with metal to form a mixed conducting interphase (MCI) that continuously grows and (c) shows the SSE decomposing to form an ionically conducting interphase (or solid electrolyte interphase, SEI), which remains stable after formation. (d) SSEs such as LLZO are kinetically stabilized with Li metal but do not wet fully. (e) Additional lithophilic coatings can be applied by ALD or PLD followed by heating with the metal to form a reactively wetted interface. Figure adapted from Ref. [100]. Copyright 2015 Elsevier.

Two types of interphase formation at the metal anode/SSE interface can be defined based on the thermodynamic driving force of the reaction to form a new interphase(s) (Δ*G*) and the electronic conductivity of the phases formed (Figure 8).[^99^, ^100^] These are:

1. Intrinsically stable interfaces (Δ*G* >0). Very few true examples exist for the pure parent alkali metal/SSE couple in battery systems, with a possible exception being Na‐β‐alumina and Na metal. Most binary oxides such as LiX (X=Cl^−^, Br^−^, I^−^, F^−^), Li_3_N and Li_3_P are stable with Li metal and exhibit no spontaneous chemical decomposition.^[28]^ The LLZO/Li interface is a special case in which the interface is kinetically stabilized due to the very low driving force for the reaction (see Section 3) Some reports have also suggested that further stabilization of the interface may be due to lithiation of surface layers of LLZO, forming a stable tetragonal interphase.^[101]^ At these interfaces, adhesion is described by nonreactive wetting. Other examples include interfaces with metallic alloys, such as Li‐In, in which the thermodynamic driving force for electrolyte reduction is lowered compared to the pure Li metal, leading to a stable interface with certain electrolytes.^[102]^
2. Thermodynamically unstable interfaces (Δ*G* <0) in which a new phase or phases are formed at the interface. These can be further subdivided into two cases:
3. Formation of a mixed ionic electronic conducting interphase (MCI). These types of interphases are unstable because both ionic and electronic conductivity contribute to continuous decomposition of the interface. For example, some NASICON‐type SSEs fall under this category (e. g., LATP and LAGP). Artificial protection of sulfides is often used to prevent them from being oxidized in this way too. At these interfaces, reactive wetting is continuously occurring.^[103]^
4. Formation of an ionically conductive but electronically insulating solid electrolyte interphase (SEI). The interphase, once formed, passivates the interface and becomes kinetically stabilized. Examples include LiPON electrolytes, which form a nanometrically thin interphase with Li metal.^[104]^ At these interfaces, reactive wetting changes to nonreactive wetting. Providing the interphase formed has good ionic conductivity, this is the desirable situation that we are looking for in a reactive wetting scenario. Understanding the adhesion between Li metal and the newly formed interphase is crucial.

Theoretical calculations (Section 3) predict strong wetting for several metal/SSE couples (e. g., the pure Li/LLZO interface).[^105^, ^106^, ^107^] However, this is not always what we observe experimentally. In the case of Li/LLZO, this is exemplified by numerous reports of high and differing values of interfacial resistance (usually reported as an area specific resistance, *ASR*). High interfacial resistances for the Li/LLZO system often originate from the non‐native phases at the interface being measured that can introduce a higher resistance to ion transport and/or decrease the electrochemically active area due to poorer wettability at the interface (see Section 4: Experimental strategies to control wetting for an overview of these values and mitigation strategies for this interface).[^11^, ^23^, ^33^, ^43^, ^80^, ^108^, ^109^, ^110^] This highlights the highly surface‐sensitive nature of wetting, and the necessity of knowing the outermost surface composition precisely of the material under study.

#### 2.2.2 Dynamics of the SSE/metal anode interface

##### 2.2.2.1 Charge transfer (or alkali metal stripping and plating) at the SSE/metal anode interface

**Figure 9:**
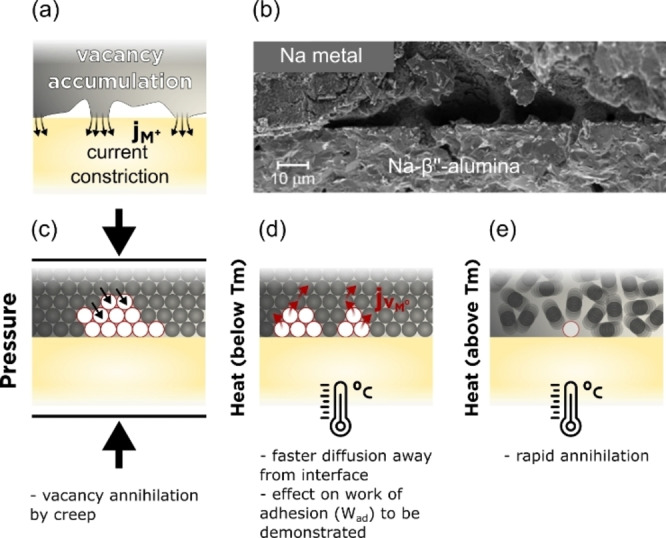
Evolution of metal/SSE interface: (a) when vacancy accumulation occurs forming voids leading to contact loss; (b) cross‐section SEM images illustrating void coalescence at the Na/Na‐β’’‐alumina interface. Reproduced with permission from Ref. [112]. Copyright 2020, American Chemical Society. Physical solutions to maintain the integrity of the interface include: (c) improving metal creep by applying pressure to the metal anode; (d) increasing the cell operating temperature; (e) going above the melting point (*T*_m_) of the metallic electrode.

The picture becomes more complex when we consider the interface under dynamic conditions, such is the case for an alkali metal/SSE interface, which sets it apart from other conventional metal/ceramic adhesion problems. During discharge, an alkali metal ion (*M* ^+^) crosses the alkali metal/SSE interface to a vacant or interstitial site in the near‐surface SSE, leaving an electron, e^’^(*M*), and a vacant site, V^×^_M_(*M*), in the metal electrode surface. The created metal vacancy, V^×^_M_(M), has to move away from the interface by diffusion. The calculated sum of the vacancy formation energy and activation energy at room temperature is relatively large for lithium metal at around 0.54 eV, which creates a concentration gradient of vacancies in the metal.^[111]^ Krauskopf et al. suggest that if the stripping current surpasses the metal diffusion dissolution limit, the interface morphology will destabilize and vacancies will coalesce leading to void formation at the interface.^[87]^ This will be enhanced by the domination of adatom diffusion along the surface of pores over vacancy diffusion in the bulk metal, which leads to fast growth of voids that tend to accumulate (Figure 9a). Note that even with relatively low current densities, for example 2.5 mA cm^−2^ every second, Li metal depletes in thickness by 3.37 nm (6.13 nm for Na). At high current densities required for fast charging applications (>10 mA cm^−2^) the depletion of 10 s of nm of Li metal from the interface every second becomes a significant challenge.

The contact loss due to void formation at the alkali metal anode/SSE interface leads to several problems such as dendrite nucleation and growth that has been associated with the creation of hotspots with highly localized pressures and overpotentials.[^32^, ^33^, ^87^] Recent in situ X‐ray computed tomography coupled with spatially mapped X‐ray diffraction studies suggest that localized current densities facilitate crack nucleation at the metal/SSE interface that afterwards grow towards the opposite electrode opening paths for dendrite propagation.^[113]^ Retaining good contact at the alkali metal/SSE interface is therefore paramount and strategies to quantify its evolution and optimize its performance are urgently required. Experimentally, the formation of voids can be followed electrochemically due to the associated increase in interfacial resistance (constriction resistance).^[33]^ This constriction resistance can be minimized by controlling the mechanical properties of the alkali metal through optimization of external parameters such at temperature and pressure.[^33^, ^112^, ^114^, ^115^]

The application of external pressures leads to a deformation of the Li (or Na) metal through mechanical creep, which involves the motion of dislocations within the alkali metal, that drives Li atoms to the metal/SSE interface (Figure 9c). Void formation is therefore predicted to occur on stripping when the flux of Li^+^ into the electrolyte (*J*_Li+,SSE_) is greater than the flux of Li atoms moving towards the interface via both bulk diffusion (*J*_Li,bulk_) and creep (*J*_Li,creep_), i. e. *J*_Li+,SSE_>*J*_Li,bulk_+*J*_Li,creep_.[^32^, ^114^] The effect of pressure was demonstrated in an Li/LLZO/Li cell, where negligible interfacial resistances were obtained at 400 MPa of external preforming pressure.^[33]^ During stripping experiments, a reduced pressure of 35 MPa was needed to retain good contact and prevent void formation. Under low applied pressures of 4 MPa, significant void formation was observed upon extended cycling of the Na/Na‐β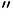 alumina interface at a current density of 1.5 mA cm^−2^ (Figure 9b).^[112]^ Pressures above 7 MPa were required to suppress void formation at 1.5 mA cm^−2^ and pressures greater than 9 MPa were required at a current density of 2.5 mA cm^−2^. Despite its effectiveness, the application of high external pressures is not viable in many of the practical applications where SSB will be used such as in electric transport.

The metal mechanical properties can also be improved by increasing the working temperature. An increase in the critical current density for cell failure was observed for both Li/LLZO^[114]^ and Na/Na‐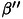 alumina^[11]^ as the temperature of the systems was increased, below the melting point of the alkali metal. The increase in the critical current density was ascribed to the enhanced alkali metal transport to the metal/SSE interface helping to suppress void formation Figure 9d. A dramatic increase in the critical current density for cell failure is observed when the alkali metal is heated into the molten state.[^12^, ^116^] The use of a liquid metal anode fundamentally suppresses void formation at the alkali metal/SSE interface due to the rapid annihilation of metal vacancies in the liquid (Figure 9e). Current densities up to 530 mA cm^−2^ have been achieved at the Li/LLZO interface at 195 °C before dendrites are formed,^[116]^ whereas current densities of 2600 mA cm^−2^ were possible for the Na/Na‐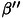 alumina interface at 250 °C. However, the use of high temperatures and containment of highly reactive liquid alkali metals remains a key challenge for practical applications.

##### 2.2.2.2 Role of grain boundaries in dendrite nucleation

**Figure 10:**
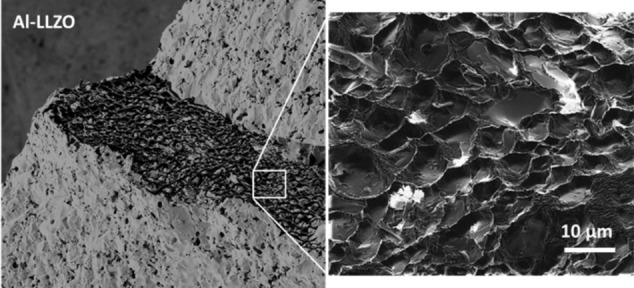
SEM images of cleaved Al‐doped LLZO pellet showing Li dendrite propagation along grain boundaries. Reproduced with permission from Ref. [117].

The polycrystalline nature of typical SSE ceramic materials means that grain boundaries play a significant role in the performance and failure modes of SSBs. Their unique physical and chemical properties, which differ from the bulk, can affect the behavior of dendrites nucleation and propagation material, often facilitating the growth of a dendrite network during cycling (Figure 10).^[117]^ Studies have shown that alkali metal dendrite growth can occur preferentially in the grain boundary (GB) regions of polycrystalline ceramic electrolyte, such as LLZO, even when the grain interior (GI) regions have higher ionic conductivity.^[118]^ Both the ohmic overpotential (ionic conductivity‐dependent), and the kinetic overpotential (which depends on the effective stress‐induced current density at the lithium/electrolyte interface) need to be overcome for deposition to occur in grain boundaries.

Monroe and Newman demonstrated how the electrochemical potential is impacted by both the mechanical stress experienced by the Li metal and SSE, and the molar volume of Li metal.^[119]^ Under elastic deformation, the stress in the Li and SSE is a linear function of the elastic modulus. As demonstrated by Barai et al., the effective stress induced current density is exponentially related to the change in the electrochemical potential through a modified Butler Volmer type relationship.^[120]^ The elastic moduli of GB's are typically lower than mechanically stiffer GI, which leads to a smaller change in the electrochemical potential in the former case, and thus a higher effective stress induced current density at the GB.^[120]^ The higher stress induced current density of the mechanically softer GBs leads to enhanced lithium deposition in the GB and subsequent dendrite growth.[^120^, ^121^, ^122^]

As described by Li and Monroe,^[123]^ if the free energy cost for deposition of Li at the Li/SSE interface is too high, then it becomes more favorable to deposit at the grain boundaries (dendrite nucleation along the grain boundaries, also viewed as the formation of a new intergranular phase). In this work, the stress associated with dendrite growth was shown to be much lower than the fracture stress of the SSE,^[123]^ an observation shown previously for sodium beta‐alumina.^[124]^ Dendrites could thus nucleate whenever an applied current induces a particular critical pressure, Δ$p_{c}$ , (the difference between the pressure experienced in the bulk and at the surface). Most ceramic electrolytes have capacitive interfaces with a metal electrode, so sustain space‐charges under pressure, meaning that an applied voltage causes the bulk electrolyte to feel tensile stress relative to the surface. The compressive stress felt at the Li/LLZO interface makes the free‐energy cost of interfacial deposition high enough that it becomes more favorable to deposit Li in the grain boundaries. They demonstrated that the critical pressure, Δ$p_{c}$ is proportional to the difference in energy between the LLZO grain boundary energy ($\gamma_{LLZO/LLZO})\;$ and twice the Li/LLZO interfacial energy ($\gamma_{Li/LLZO}$ ) as: Δ$p_{c}\propto \gamma_{LLZO/LLZO}-$ 2$\gamma_{Li/LLZO}$ , where the factor of 2 arises on the right had side due to the fact that two new Li/LLZO interfaces are formed during dendrite nucleation in the grain boundary. They highlight that a possible strategy to suppress dendrite nucleation in the grain is to reduce the interfacial adhesion between Li metal and the SSE (i. e. increase $\gamma_{Li/LLZO}$ ), however, as will be discussed in Section 3.2.5.2 and Section 4, strong metal/SSE interfacial adhesion (small $\gamma_{Li/LLZO}$ ) is crucial for the suppressing void formation in SSBs with alkali metal anodes. This paradox between maintaining strong alkali metal adhesion at SSE surfaces but avoiding alkali metal adhesion in grain boundaries illustrates the importance of considering the complex interplay between both microstructure and chemistry of both grains and grain boundaries in relation to the failure mechanisms of ceramic electrolyte‐based cells.

### 2.3 Spectroscopy of surfaces and interfaces

As discussed in Section 2.1.4, the surface chemistry of SSE materials plays a critical role on the nature of interfacial adhesion with alkali metals, and so accurate characterization of SSE surface chemistry is essential to design interfaces that will be stable in practical SSB systems. A diverse range of advanced characterization techniques have been developed over the last 20 years for studying SSBs, both in situ and ex situ, and the reviewers are directed towards the following reviews for an in‐depth discussion on the topic.[^125^, ^126^, ^127^, ^128^, ^129^] In the following section, we provide a brief background to selected characterization techniques that have been utilized to probe the surface and interfacial chemistry of SSE materials, with the goal of understanding and improving the interfacial adhesion in practical devices.

#### 2.3.1 Characterization of surfaces

**Figure 11:**
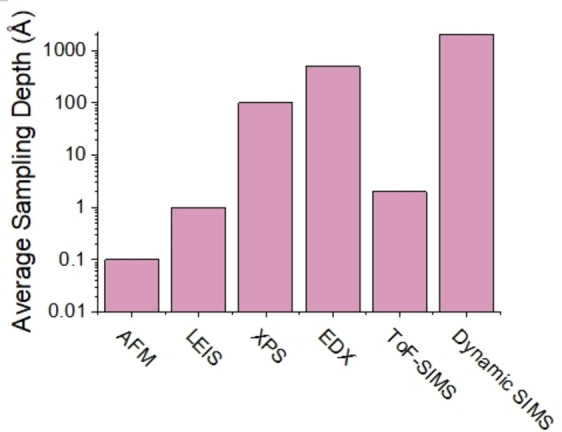
Average sampling depth of different surface analysis techniques. Sampling depth values taken from Ref. [130].

A key characteristic of different surface analysis techniques is their sampling depth resolution and destructive versus nondestructive nature. The range of depth resolution spans from analysis of the first monoatomic layer, with techniques such as low energy ion scattering (LEIS) and atomic force microscopy (AFM), to the near surface region with X‐ray photoelectron spectroscopy (XPS), to subsurface and bulk regions using energy‐dispersive X‐ray spectroscopy (EDX) and dynamic secondary ion mass spectrometry (SIMS), as shown in in Figure 11.^[130]^

In the study of surfaces of SSBs, very high depth resolution is required. LEIS is a nondestructive technique that measures the change in energy of a scattered nobel gas ion at a specific angle, to give quantitative elemental analysis of the composition of the first atomic layer of a surface. This technique has been used to understand the nature of surface terminations in NASICON electrolytes that have improved wettability with Na metal^[131]^ and to quantify surface coverage by contaminant layers in garnet electrolytes.^[80]^ In an XPS measurement, the surface of a material is irradiated with X‐rays from a laboratory or synchrotron‐based source under ultra‐high vacuum conditions, which allows the elements present in the first few nanometers of a materials surface and their chemical bonding environments to be probed. XPS has been widely utilized for studying the presence of contaminant phases (see Section 4.2), formed on as‐synthesized materials, such as carbonate layers on the surface of LLZO.^[43]^ In situ and operando XPS techniques have also been developed in which changes in the surface composition of SSE materials can be characterized during the in situ chemical or electrochemical deposition of alkali metals.[^132^, ^133^, ^134^] XPS can provide important information on the chemical composition of SSE and interlayer surfaces and contaminant layers, however, it has limited lateral resolution which prevents a detailed mapping (either 2D or 3D) of surfaces, and so it is often coupled with other techniques when analysis of the spatial chemical distribution is required.

**Figure 12:**
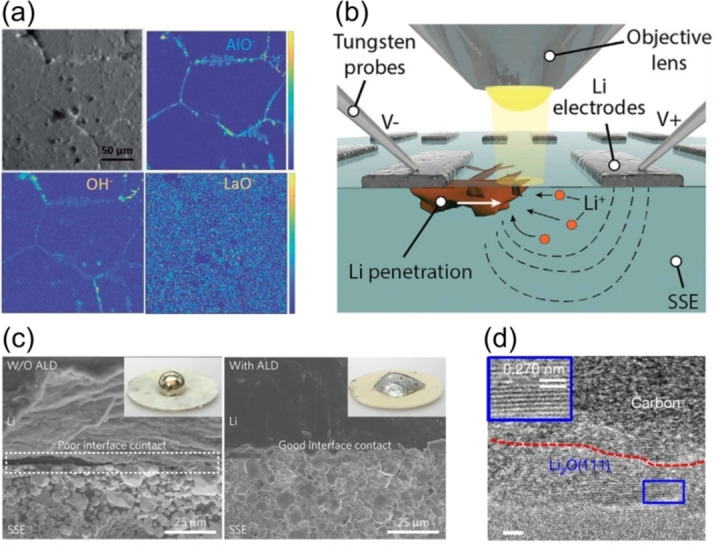
(a) ToF‐SIMS analysis of surface of thermally etched LLZO pellet showing the segregation of Al and H species to grain boundaries. Reproduced with permission from Ref. [135]. Copyright 2021 Royal Society of Chemistry. (b) In situ optical spectroscopy setup for studying in situ Li metal penetration in a SSE. Reproduced with permission from Ref. [136]. Copyright the Authors 2020. Published by Elsevier Inc. (c) Cross‐sectional SEM images of SSE/Li metal interface showing ‘poor’ and ‘good’ interfacial contact in the absence and presence of a Al_2_O_3_ interlayer, respectively. Reproduced with permission from Ref. [76]. Copyright 2016, Nature Publishing Group. (d) TEM image showing Li_2_O layer formed in situ on the surface of a hollow carbon tubule. Reproduced with permission from Ref. [137] Copyright 2020, The Author(s), under exclusive licence to Springer Nature Limited.

SIMS is a powerful technique that can be used to determine the spatial distribution of elements on surfaces. In a SIMS measurement, the surface of a sample is irradiated with a primary ion beam, resulting in the ejection of secondary ions that are collected for detection. Depending on the SIMS detection mode and characteristics of the primary beam, depths from a few surface monolayers, all the way to the bulk can be probed (Figure 11). In time‐of‐flight secondary ion mass spectrometry (ToF‐SIMS), low energy primary ions are used to give detailed chemical information of the top few atomic layers. In dynamic SIMS mode, higher energy primary ion beams are used to sputter the sample, allowing chemical analysis deeper into the bulk to take place. Using ToF‐SIMS, information about all chemical species present on the surface are collected simultaneously from across the periodic table, creating a full chemical map of the material. An example of this was the recent study by some of the present authors in which ToF‐SIMS was utilized to understand the role of thermal etching on the wettability of lithium on LLZO electrolytes (Figure 12a).^[135]^ Upon thermal etching, both disappearance of surface contaminant layers and the segregation of elements such as Al and H to the grain boundaries was captured by ToF‐SIMS, which has important implications for the reactivity and wettability with Li metal.

#### 2.3.2 Characterization of interfaces

The analysis of pristine surfaces is of pivotal importance to assess and predict the behavior of materials before assembly into a device. However, techniques to probe the buried interface between SSEs and the alkali metal anode are also of great importance to understand changes in the interfacial structure and interfacial adhesion during electrochemical cycling. A range of optical microscopy‐techniques have recently been developed to study the evolution of the SSE/Li metal interface. In a recent publication, Kazyak et al. employed operando video microscopy to investigate lithium penetration in several ceramic solid electrolytes (Figure 12b).^[136]^ They used an in‐plane cell geometry and carried out high quality optical operando imaging of SSEs at battery‐relevant current densities in order to show how lithium penetrates with multiple different morphologies in LLZO and LPS electrolytes. They stressed the importance of considering the impact of both plating and stripping on the Li/SSE interface by observing the morphology evolution of the Li electrode which leads to void formation and dewetting during cell cycling. Although video microscopy has proven to be a powerful technique for studying interfaces and the propagation mechanism of lithium in SSEs, its resolution on the micron scale can limit the amount and quality of information that can be extracted. Enhanced resolution can be obtained by moving from visible light to X‐ray based radiation sources. An example of this was in a recent study in which X‐ray computed tomography coupled with spatially mapped X‐ray diffraction was used to visualize dendrite propagation, proving that crack nucleation and propagation at the Li/Li_6_PS_5_Cl/Li interface precedes dendrite propagation.^[113]^

Higher resolution down to the nanometer scale can be obtained by using electron microscopy (EM), specifically secondary or transmission electron microscopy (SEM and TEM). SEM microscopy is routinely used to visualize the degree of interfacial contact between SSE materials and alkali metals, in addition to the morphology of interlayers, typically by taking cross‐sections through planar SSE/alkali metal cells. An example of this was in the study by Han et al. in which cross‐sectional SEM was used to investigate the improved adhesion between Li metal and garnet SSE interfaces through the addition of thin Al_2_O_3_ interlayers (Figure 12c).^[76]^ Operando electron microscopy has been recently used for a real‐time investigation of the various phenomena occurring at interfaces. For example, Chen et al. demonstrated that alkali metals display homogeneous stripping and plating on ZnO_*x*_‐coated hollow carbon tubules (Figure 12d).^[137]^ The enhanced electrochemical deposition of Li metal within the tubules through a diffusional Coble creep mechanical was attributed to the enhanced wettability of the Li metal with ZnO_x_, which forms a Li_2_O layer in situ.

Although electron microscopy techniques are amongst the most powerful techniques to investigate interfaces, it is worth noting that the electron beam can have detrimental effects on the stability of the battery components, resulting in atomic displacement and e‐beam sputtering caused by elastic scattering and heating/contamination/damage of the sample due to inelastic scattering.[^138^, ^139^] This has to be taken into account when assessing the imaging processes, and precautions such as reducing the electron dosage should be adopted. In this regard, cryo‐TEM is particularly useful, especially on the study of metallic Li/SSE interfaces, and fabrication of TEM lamellae can be achieved by considering the fine balance between the chemical, thermal, electrical and mechanical properties of all the cell components.^[140]^ Lee et al. have recently used cryo‐TEM in order to characterize the Li/LiPON interface. Particular focus was directed to how the electrolyte morphology can impact the nucleation, density and morphology of plated metallic lithium, and therefore the cycling performances of SSE based cells.^[140]^ Similarly, Wang et al. have also used cryo‐TEM to study nucleation and growth of metallic lithium, suggesting that glassy Li outperforms crystalline Li in electrochemical reversibility, making it desirable for high‐energy rechargeable batteries.^[141]^

## 3 Computational Modeling of Interfaces

Computational modeling has proved to be an invaluable tool across the battery field for understanding the fundamental processes that occur from the atomistic scale up to the full battery pack level. Whilst cutting edge characterization techniques continue to be developed, probing the materials chemistry of buried interfaces in ASSBs at the atomistic level continues to be a significant challenge experimentally. Ab initio density functional theory (DFT) based models have been extensively used to investigate the atomistic structure,[^16^, ^142^] stability,[^105^, ^143^] synthesisability,[^144^, ^145^, ^146^] and conductivity[^147^, ^148^] of bulk solid electrolyte materials with near quantum chemical accuracy. Over the last 5 years, increasing efforts have been directed towards using DFT calculations to understand the interfacial structure and reactivity between solid components in ASSBs. Alongside these efforts, progress has also been made on the development of multiscale models to link the atomistic phenomena at interfaces to the mechanical and transport properties at the particle, or even cell levels.^[149]^ In the following sections, an overview of recent computational methods used to understand the local structure of interfaces and nature of wetting in ASSBs is given.

### 3.1 Interfacial stability (bulk phases)

As highlighted in Section 2.2.1, the electrochemical stability window of a bulk SSE dictates whether the material will be stable against the reducing and oxidizing environments at the anode and cathode, respectively. The applied voltage, *V*, that the SSE experiences can be related to the chemical potential of the alkali anode, such as Li ($\mu_{Li})$ through the relationship given by Eq. 12:

(12) $$\mu_{Li}=\mu_{Li}^{0}-eV$$

where $\mu_{Li}^{0}$ is the chemical potential of bulk Li metal.^[150]^ The thermodynamic driving force for two materials to react can be assessed from the grand potential phase diagram that contains all chemical species in both phases.[^105^, ^151^, ^152^] To form a grand potential phase diagram, the energies of all stable bulk phases within the compositional space needs to be known in advance. The construction of large databases of DFT calculated energies of bulk structures, such as the Materials Project,^[153]^ and the Open Quantum Materials Database,^[154]^ allows for the rapid calculation of grand potential phase diagrams and the assessment of interfacial stability of SSE materials against both the anode and cathode for ASSB.

**Figure 13:**
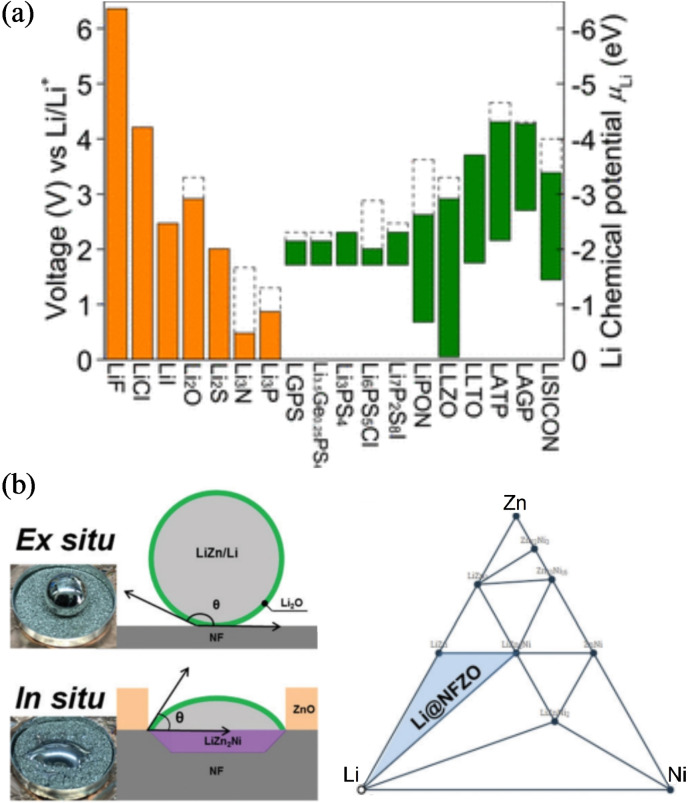
(a) Electrochemical window for different Li solid‐electrolyte materials (green) and decomposition products (orange), calculated using DFT energies from the Materials Project Database. Reproduced with permission from Ref. [143]. Copyright 2015 American Chemical Society. (b) Reaction between LiZn alloy and nickel foam (NF) forming LiZn_2_Ni alloy in situ. The corresponding ternary Li‐Ni‐Zn phase diagram predicted from first principles is shown. Adapted with permission from Ref. [155]. Copyright 2020 Royal Society of Chemistry.

This approach has been successfully used to probe the stability of Li (Figure 13a) and Na metal against different classes of solid electrolyte materials.[^105^, ^143^, ^156^] DFT analysis predicts a large electrochemical window (2.86 V) for Li_7_La_3_Zr_2_O_12_ garnet (LLZO) with a very small driving force (−0.021 eV atom^−1^) for decomposition into Zr, La_2_O_3_ and Li_2_O against Li metal, which is consistent with the experimentally observed stability of LLZO.[^143^, ^157^] In contrast, sulfide materials, such as Li_3_PS_4_, have a very narrow stability window (0.60 V) and a large driving force (−1.42 eV atom^−1^) for decomposition against Li metal via the reaction Li_3_PS_4_→Li_2_S+P.^[143]^

The thermodynamic driving force for the reaction between Li metal and other components of the ASSB is an important criterion for reactive wetting, as outline in Section 2.2.1. Assuming that the entropy of solid phases is small, the change in free energy ΔG of a reaction between two components in Eq. (8), can be approximated as the thermodynamic driving force from the grand canonical phase diagram as described above. In recent years, first principles phase diagrams have been used to understand the nature of the phases that form during alloying reactions. Wang et al. used DFT energetics from the Materials Project database to demonstrate how a larger Gibbs free energy for the reaction between Li metal with coating materials (Au, Ag, Al, ZnO, TiO_2_ and Al_2_O_3_), correlates with a smaller experimentally observed contact angle of liquid Li metal droplets on the respective substrates.^[75]^ Au was found to have the largest Gibbs reaction energy of the pure metals investigated computationally, which was consistent with the smallest contact angle observed experimentally.

In a seminal study by Fu et al., first principles phase diagrams, in conjunction with experimental techniques were used to understand the reaction energy between Li metal and a thin film of Al metal added to the surface of an LLZO garnet material to improve wettability.^[158]^ Large reaction energies on the order of 40–60 meV atom^−1^ were predicted for the formation of Li_*x*_Al alloy phases. In a more recent study, first principles phase diagrams were used to understand the interaction between Li metal, ZnO and a Ni foam alloy.^[155]^ The in situ formation of an intermetallic LiZn_2_Ni phase, as predicted on the computational phase diagram (Figure 13b), was found to be crucial to facilitate the wetting of LiZn on the surface of the Ni foam.

### 3.2 Explicit surface and interface calculations

The calculation of thermodynamic properties using bulk phases with infinitely periodic cells provides a quick and efficient method of predicting the thermodynamic driving force for reactions between materials and likely decomposition products, however, it does not take into account the changes in the local structure and bonding at a materials surface and at the interface between two solid materials. As will be discussed in the following sections, by explicitly including the presence of surfaces/interfaces in the DFT calculations, a deeper understanding of the local phenomena that lead to wetting can be gained and used to rationalize experimental data.

#### 3.2.1 Surface energy calculations

The surface energy ($\sigma_{s,vac}$ ) of a solid material (see Section 2.1.1) along a specific crystalline facet of Miller index *hkl* can be calculated by Eq. 13:

(13) $$\sigma_{s,\mathrm{vac}}=\frac{1}{2A}\left(E_{s}^{\mathrm{surf}}-E_{s}^{\mathrm{bulk}}-\sum_{i}^{\mathrm{species}}\Delta n_{i}\mu_{i}\right)$$

**Figure 14:**
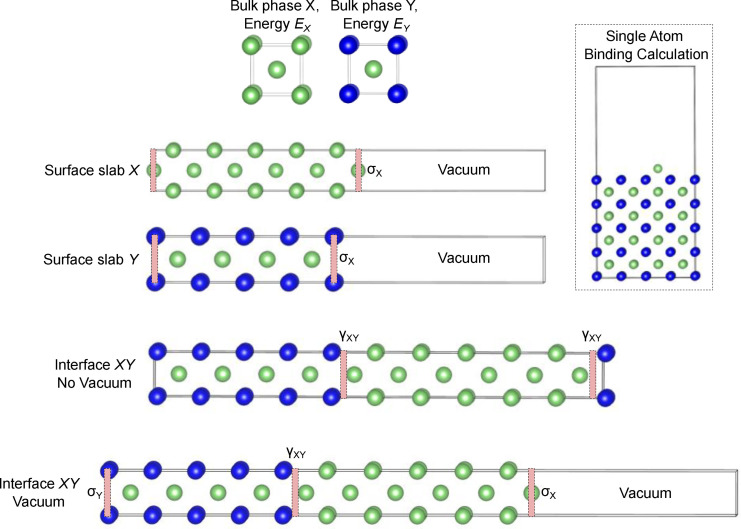
Schematic depiction of bulk, slab, and interface geometries used in DFT calculations. Pink dashed areas indicate surface or interface planes of area A, with energies of σ and γ , respectively. Dot‐dash box shows schematic depiction of single atom binding calculation on surface of phase Y.

The free energy of the materials surface, $E_{s}^{surf}$ , is evaluated with DFT calculations in which a slab with cross‐sectional area *A*, consisting of several atomic layers of the material perpendicular to the *hkl* plane of interest are separated by a vacuum region (Figure 14).[^159^, ^160^] The energy of the corresponding bulk crystal, $E_{s}^{bulk}$ , is calculated in a periodic cell without vacuum. For nonstoichiometric surfaces, i. e. ones that have a different stoichiometry to the bulk,$\;\sigma_{s,vac}\;$ will depend on environment as dictated by the chemical potential ($\mu_{i}$ ) of species *i*, which has an off‐stoichiometry of Δ*n_i_* . Appropriate ranges for $\mu_{i}$ depends on the chemical potentials of species *i*, in solid or gaseous reference phases within the same chemical compositional space, which are also calculated with periodic DFT calculations or taken from experimental tables.[^159^, ^160^]

**Figure 15:**
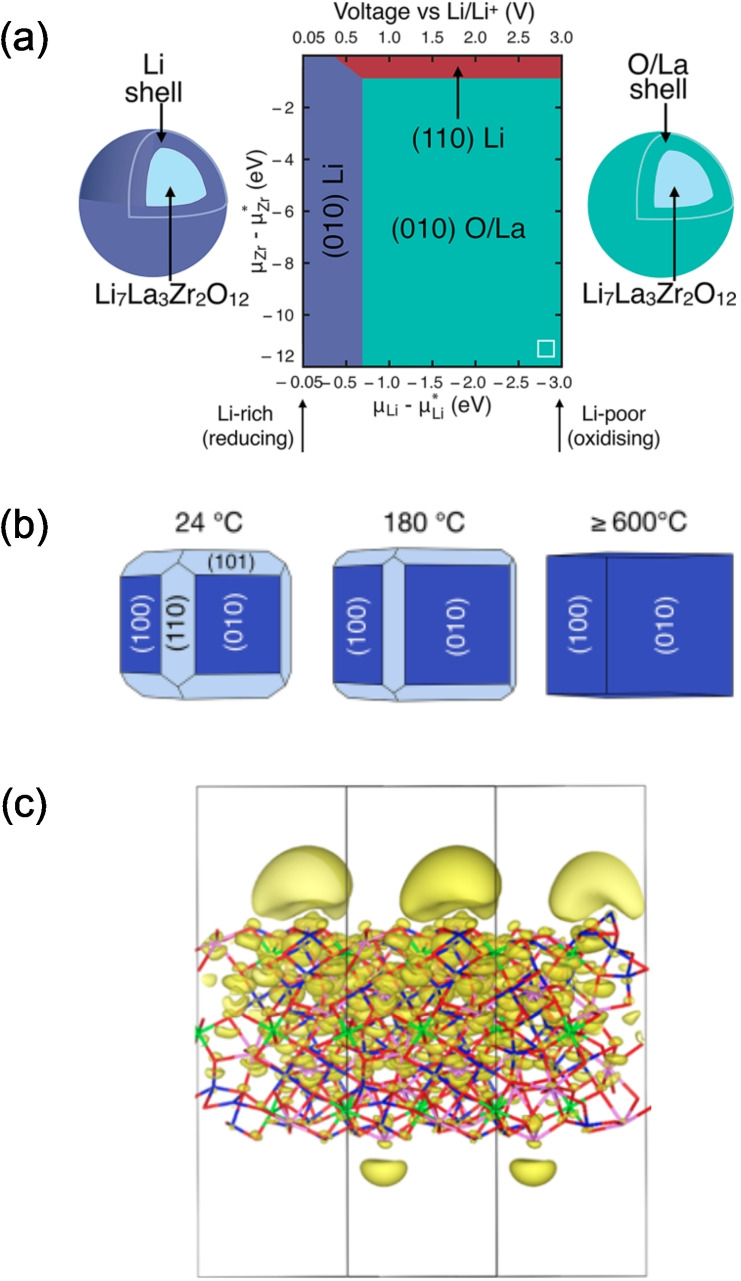
(a) Surface energy diagram of Li_7_La_3_Zr_2_O_12_ (LLZO) as a function of Li and Zr chemical potential (μ ). Reprinted with permission from Ref. [162]. Copyright 2018 American Chemical Society. (b) Wulff shape of LLZO particles as a function of temperature, showing the preference for Li‐rich (100) facets at higher temperatures. Reprinted with permission from Ref. [162]. Copyright 2018 American Chemical Society. (c) Enhanced electron density (yellow) on the (110) surface of LLZO. Adapted with permission from Ref. [166]. Copyright 2019 American Chemical Society.

By enumerating the energy of different surface terminations, the Wulff shape of a particle (see Section 2.1.2) can be predicted from first principles. First principles Wulff plots have previously been used to understand the shape of pure metals including Li and Na,[^36^, ^161^] SSE materials such as LLZO^[162]^ and NASICON (Na_4‐*x*_Zr_2_Si_3‐*x*_P_*x*_O_12_)^[131]^ and transition metal oxide cathodes such as LiNi_1‐*y*‐*z*_Mn_*y*_Co_*z*_O_2_.^[163]^ Changes in the chemical potential of the environment, such as the oxygen partial pressure and temperature during synthesis, can have a significant impact on the stability of different surface facets in solid electrolyte materials. The role of chemical potential and chemical composition on the surface structure of LLZO has be studied by several authors.[^29^, ^162^, ^164^] Under reducing (Li‐rich, O‐poor) conditions (Figure 15a) and high temperatures (Figure 15b), the surfaces of LLZO particles are predicted to show Li enrichment which may affect the tendency of LLZO to nucleate dendrites during electrochemical cycling.^[162]^

Several DFT studies have shown that changes in the local bonding at surfaces in solid‐electrolyte materials such as Li_7_La_3_Zr_2_O_12_ and Li_2_PO_2_N can affect the positions of the valence and conduction band relative to the bulk material, which can have important implications for the electrochemical stability.[^164^, ^165^, ^166^] In a study by Tian et al. it was demonstrated that there is a localization of electron density (Figure 15c) at the (110) surface of Li_7_La_3_Zr_2_O_12_, which may accelerate the nucleation of dendrites in comparison to the (100) surface of Li_2_PO_2_N which showed considerably less electron trapping.^[166]^

#### 3.2.2 Single atom binding calculations

To probe the intrinsic interaction of alkali metal atoms with different material surfaces, several authors have investigated the binding energy of a single atom (or cluster) to a materials surface using the slab models as described above (Figure 14). Single atom binding calculations have primarily been used to study the binding and nucleation of Li on lithophilic scaffold materials in liquid electrolyte systems, such as doped carbons, although they are also applicable for studying the strength of binding of metal atoms with SSE materials and current collectors, which is particularly important for the development of ‘anode‐free’ SSBs.

Doping N into the graphene structure was found to increase the binding of a Li atom compared to undoped graphene or a Cu surface.[^167^, ^168^] The increased lithophilicity of the N‐doped graphene surface was highlighted as a key factor that led to a reduction in the nucleation barrier for metallic Li in liquid electrolyte cells containing a N‐doped graphene matrix, which facilitated the uniform plating of Li and suppression of dendritic structures.[^167^, ^168^] Recently, Li‐atom binding calculations have shown that the interaction between Li and carbon structures can be further enhanced by using electronegative heteroatom dopants with strong charge transfer, such as O.^[169]^ These results are in line with the increased Li‐atom binding and improved Li plating observed in reduced graphene oxide^[170]^ and MXene/graphene oxide composite systems^[171]^ in liquid electrolyte cells.

Lithiophilic 3D porous metal current collectors are a promising avenue to suppress alkali metal dendrite formation in liquid systems and, as detailed further in Section 4.5, are promising candidates for SSE batteries as well. Single atom binding calculations were recently used to screen a wide range of metal and metal alloy materials as potential current collectors to facilitate uniform Li growth.^[172]^ All of the metallic current collectors studied were found to display strong adsorption of Li atoms (i. e. a stronger binding than Li with itself) due to the strong bonding at metallic surfaces (see Section 2.1.4.1). Li alloys such as Li‐Al, Li‐Zn and Li‐B were found to be particularly promising candidates for current collectors in comparison to conventional metals such as Cu and Ni, as they showed the optimal balance between Li binding and Li transport on the metal surface. In a separate study, single Li atom binding energy calculations were used to show that CuZn alloys are also promising 3D metallic hosts for Li deposition. Li atoms bind to Zn sites on the CuZn (111) alloy surface stronger than on Zn (101) or Cu (111) surfaces.^[173]^ Single atom binding calculations were also recently used to demonstrate how doping of Ag into the Li metal anode increases the binding energy for Li atoms, resulting in more uniform deposition of Li metal.^[61]^

Binding calculations have been recently used to understand the adhesion between solid‐polymer electrolytes and Li metal.[^174^, ^175^] The interaction of individual monomers or short chain oligomers of different types of solid‐state polymer electrolytes, such as polyethylene oxide (PEO), with the (100) surface of BCC Li metal was studied. Strong adsorption was observed for polymers systems that contained ester, carbonate or nitrile groups, however, significant reactivity and bond cleavage was also observed, particularly for systems with ester and carbonate groups.^[151]^ There have only been limited studies on alkali metal/polymer SSE interfaces to date, but as highlighted further in Section 4.3.2, polymers are a promising class of materials for interlayers in SSBs, and so further computational studies of the fundamental interfacial binding in these systems are urgently needed to guide materials design.

Single atom (or molecule) binding calculations are useful for comparing the relative binding energies of metal atoms, such as Li, with different substrate surfaces, however, they do not take into account the interaction of Li with adjacent Li atoms, which is important for understanding the structure and dynamics of the true interface during electroplating. A more accurate treatment of the interfacial structure can be gained by modeling the interface between surface slabs of alkali metals and SSE explicitly, as will be discussed in the following section.

#### 3.2.3 Explicit interface calculations

##### 3.2.3.1 Interfacial energy and work of adhesion

The interfacial structure and energy $\gamma_{XY}$ between two solid materials, *X* and *Y*, can be computed with DFT using a supercell approach, in which slabs of *X* and *Y* are combined in the same cell (Figure 14).[^152^, ^160^, ^176^, ^177^] The two materials *X* and *Y* can either be of the same chemical composition (e. g., grain boundaries) or of different chemical compositions (e. g., electrode/electrolyte). As periodic boundary conditions are used in the majority of DFT codes, two interfaces are formed between materials *X* and *Y* per supercell, which are periodically repeated along one direction of the cell. Alternatively, in order to study a single interface per cell, a common approach is to include a vacuum region (typically >10 Å) between the components at one of the interfaces.

The formation energy (*E*_f_) for a stoichiometric interface (without vacuum) between materials X and Y, with *n_i_* formula units is defined as Eq. 14:
(14)

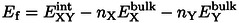




Where $E_{XY}^{int}$ is the DFT calculated energy of an interface structure formed between slabs of *X* and *Y*.^[178]^ $E_{i}^{bulk}$ is the energy of material *i* in the fully relaxed, energy minimized bulk state. For nonstoichiometric interfaces, the chemical potential of additional elements can be added, analogous to the calculation of surface energies in Eq. (13). As a single supercell structure is used for slabs *X* and *Y*, a common approximation that is adopted to limit the supercell size is the ‘coherent interface approximation’ in which the lattice parameters of one or both phases are strained relative to their bulk phases within the interface plane to form a coherent interface. The imposed lattice strain is associated with a strain energy, ζ . For an interface with area *A*, *E*_f_ can written as the sum of the interfacial energy, $\gamma_{XY}$ , and strain energy ζ [Eq. 15:[^178^, ^179^]
(15)

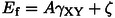




The contributions of $\gamma_{XY}$ and ζ to *E_f_* can be separated by different approaches such as the linear fitting procedure[^176^, ^180^] or ‘direct calculation’^[178]^ methods. In the ‘direct calculation’ method of Wolverton et al., for a supercell where the lattice parameter of phase *Y* has been strained, $\gamma_{XY}$ , is given by Eq. 16:

(16) $$\gamma_{XY}=\frac{E_{XY}^{int}-n_{X}E_{X}^{bulk}-{n_{Y}E}_{Y}^{strained}}{2A}$$

In Eq. (16),$\;E_{Y}^{strained}$ corresponds to the energy of bulk phase *Y* in which the lattice parameters in the *ab* plane of the interface have been strained to match those of the supercell, while the *c* lattice parameter is allowed to relax.^[178]^

From the supercell approach without vacuum described above, the work of separation, $W_{sep}$ (see Section 2) between materials *X* and *Y* can be calculated by Eq. 17:

(17) $$W_{sep}=\sigma_{X,vac}+\sigma_{Y,vac}-\gamma_{XY}$$

Where $\sigma_{X,vac}$ and $\sigma_{Y,vac}$ are the (relaxed or unrelaxed) surface energies of pure materials of *X* and *Y* in vacuum as calculated from DFT calculations in Eq. (13).^[1]^ For a system containing a single interface between phases *X* and *Y* with a vacuum region, the work of separation can simply be calculated as the difference in the energy between isolated surface slabs of phases *X* and *Y*, and the energy of the strained interface containing both phases [Eq. 18]:

(18) $$W_{sep}={(E}_{X}^{surf}+E_{Y}^{surf}-E_{XY}^{int})/A$$

For a chosen pair of surface terminations of phases *X* and *Y*, the atomic arrangement corresponding to the highest (lowest) value of *W*_sep_ ($\gamma_{XY}$ ) can be found in a single interface cell by minimizing the energy with respect to relative translations of the slabs in the *ab* interface plane and along the *c* axis. An elegant method to approximate the lowest energy configuration is to rigidly vary the distance between slabs of *X* and *Y* and fit *W*_sep_ from the resulting DFT energies using the Universal Binding Energy Relation (UBER).[^43^, ^62^, ^181^, ^182^] Once a low energy configuration has been located with UBER, an optimization of the atomic positions can be performed to capture relaxation or reconstruction at the interface.^[183]^

The work of adhesion (*W*_ad_) in Eqs. (4–6) is more challenging to directly calculate with DFT calculations, as the surface energies of phases *X* ($\sigma_{X,V})$ and *Y* ($\sigma_{Y,V})$ , in equilibrium with vapor are required [Eq. 19]:

(19) $$W_{ad}=\sigma_{X,V}+\sigma_{Y,V}-\gamma_{XY}$$

As outlined in Section 2.1.2 and Section 2.1.3, the contact angle $\theta_{c}$ is related to the $W_{ad}$ , (not *W*_sep_), and the surface energy of one of the solid phases such as Li metal in contact with vapor: *W*_ad_=$\sigma_{Li,V}$ (1+cos$\theta_{c}$ ). The value of *W*_ad_ can be approximated from *W*_sep_ through knowledge of the variation in surface and interface energies as a function of the vapor pressure.^[184]^ For clean surfaces under ultrahigh vacuum conditions *W*_ad_≈*W*_sep_. Although there is a fundamental difference between *W*_ad_ and *W*_sep_, across the literature, the work of separation in vacuum calculated in Eq. (17) is described as the work of adhesion, *W*_ad_. To be consistent with previous work, we will use the term work of adhesion and the symbol *W*_ad_ in the following sections to describe the interfacial adhesion calculated using Eq. (17) in previous reports, although reader discretion is advised.

##### 3.2.3.2 Modeling semicoherent, incoherent and complex interfaces

The same treatment as described above is also possible for semicoherent interfaces containing misfit dislocations, although larger supercell structures are typically required to model realistic misfit dislocation densities, which are at, or beyond the limit of current DFT capabilities.[^51^, ^185^, ^186^] Calculations of coherent interfaces are therefore often adopted to approximate the regions of coherent‐like interface in between misfit dislocations.

**Figure 16:**
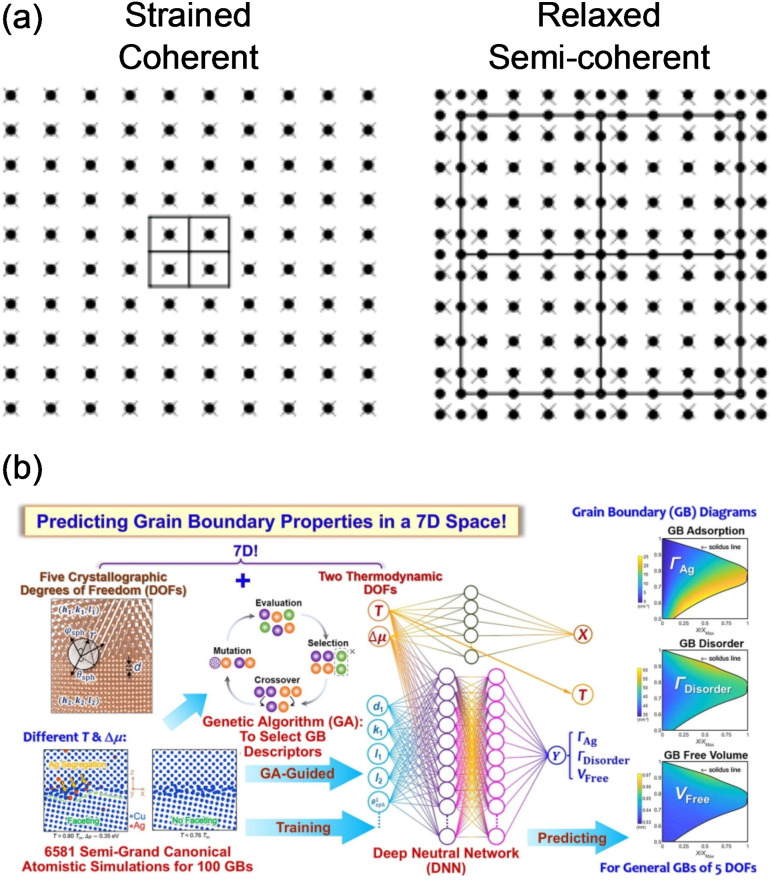
(a) Schematic representation of coherent and semicoherent interfaces between two phases, phase 1 (circles) and phase 2 (crosses). For the coherent interface, the lattice parameter of phase 2 has been strained to match phase 1. For the semicoherent interface, the atomic positions of phase 2 have been allowed to relax to equilibrium. Adapted with permission from Ref. [51]. Copyright 2002 IOP Publishing Ltd. (b) Schematic depiction of combined atomistic simulation and deep neural network machine learning model for calculation of full grain boundary diagram of a metallic material. Reprinted with permission from Ref. [197]. Copyright 2020 Elsevier.

For a coherent interface, the equilibrium values of *W*_ad_ (or *W*_sep_) in Eqs. (17–19) are well defined, as the atoms at the interface experience symmetrically equivalent sites. In the case of a semicoherent interface, the presence of misfit dislocations means that the atoms at the interface experience a range of different bonding interactions (Figure 16a).^[51]^ Early calculations on the MgO/Al (100) system, showed that the *W*_ad_ for a model coherent interface was around four times larger than that of an interface containing misfit dislocations.^[187]^ A series of strategies have since been proposed to predict the average semicoherent interfacial energy from coherent interface calculations.[^185^, ^188^, ^189^]

In the methods described above, explicit interfaces can be formed between a specific combination of surface slabs of alkali metals and ceramic or metallic materials, which are often chosen based on the low(est) energy surfaces observed in each material. In real polycrystalline ceramic systems, a distribution of surface terminations will be present that will result in a diverse range of complex interfaces with alkali metals. Predicting the structure of low energy solid/solid interfaces without experimental input remains a challenge for computational modeling, particularly at the DFT level. However, the development of structure predictions techniques in combination with novel machine learning algorithms over the last 10 years provides a promising avenue for exploring more realistic solid/solid interfaces. Techniques such evolutionary algorithms,[^190^, ^191^] particle swarm optimization[^192^, ^193^, ^194^, ^195^] and random structure searching^[196]^ have recently been applied to study the properties of complex grain boundaries and solid/solid interfaces. The ultimate computational goal would be to produce a full interface property diagram in which the energies and structures of all possible metal/ceramic surface combinations were studied. Whilst the formation of a full interface property diagram is currently unobtainable at the DFT level due to the intractable number of possible metal/ceramic surface combinations, significant progress has recently been made on the development of full grain boundary diagrams for metallic systems using a combination of computationally inexpensive embedded atom potentials and deep neural network machine learning models (Figure 16b).^[197]^

##### 3.2.3.3 Studies of alkali metal solid‐state electrolyte interfaces

Low works of adhesion have been calculated for stoichiometric interfaces between Li metal and simple ionic ceramics such as Li_2_O (*W*_ad_ *=*0.18–0.34 J m^−2^)[^176^, ^183^, ^198^] and LiF (*W*_ad_=0.07–0.09 J m^−2^).^[199]^ These materials are stable against Li metal (i. e. nonreactive wetting) and are commonly formed during the decomposition of solid‐electrolyte materials in contact with Li metal, in addition to being major components of the solid‐electrolyte interface in liquid electrolyte systems. The low work of adhesion of these materials is consistent with the weak nature of electrostatic bonding between metals and ionic ceramics outlined in Section 2.1.4.2. Larger works of adhesion have been predicted between alkali metals and electrolyte materials with enhanced covalency, such as Li/Li_3_PO_4_ (*W*_ad_=0.65 J m^−2^)[^176^, ^198^] and Na/Na_3_Zr_2_Si_2_PO_12_ (*W*_ad_=1.12 J m^−2^).^[200]^ However, the enhanced covalency of these materials means they also have large reaction energies against alkali metals (see Section 3.1) and will decompose to nonreactive binary components, such as Li_2_O, with poor wetting.

In a seminal work by Sharafi et al. a moderate work of adhesion (*W*_ad_=0.67 J m^−2^) was predicted for an incoherent interface between the (001) surface of Li metal ($\sigma_{Li}=0.45$ J m^−2^) and the (001) surface of Li_7_La_3_Zr_2_O_12_ (LLZO) garnet, corresponding to a contact angle of 62° with Li metal.^[43]^ Although LLZO is also a highly ionic ceramic with an optical band gap of 5.46 eV,^[165]^ the larger interfacial adhesion relative to Li_2_O likely stems from the electronic configuration of Zr. Previous calculations on the Ni/Al_2_O_3_ system have demonstrated that the addition of dopant transition metals with open d‐shells such as Zr and Ti lead to enhanced local bonding at the interface.^[9]^ Crucially, as indicated in Section 3.1, LLZO has very low reaction energy (0.021 eV) with Li metal and effectively remains as a nonreactive system, unlike the case of Li_3_PO_4_ described above. This result highlights that the introduction of open *d*‐shell elements such as Zr may be a promising strategy to improve the wetting of ionic ceramic materials whilst maintaining their electrochemical stability window.

**Figure 17:**
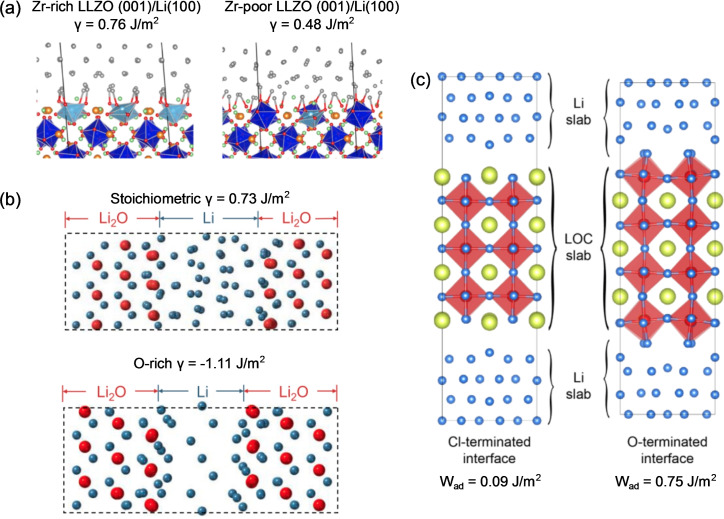
(a) Optimized interface structures between the (100) surface of Li metal and Zr‐rich and Zr‐poor (001) surfaces of Li_7_La_3_Zr_2_O_12_ (LLZO). A smaller interfacial energy (γ ) is observed for the Zr‐poor surface indicating stronger adhesion with Li metal. Adapted with permission from Ref. [29]. Copyright 2020 American Chemical Society. (b) Interfaces between the (111) surface of BCC Li metal and the (111) surface of Li_2_O for both stoichiometric and oxygen‐rich surface terminations. The large negative interfacial energy for the O‐rich system indicates significantly stronger binding than the stoichiometric configuration. Adapted with permission from Ref. [183]. Copyright 2020 American Chemical Society. (c) Incoherent interface structures between the (100) surface of BCC Li metal and the (100) surface of both Cl terminated and O terminated Li_3_OCl. Adapted with permission from Ref. [179]. Copyright 2019 American Chemical Society.

In addition to the chemical elements present in the bulk of solid electrolyte materials, it has also been shown that the surface termination plays an important role on the interfacial adhesion with alkali metals. Work of adhesion values ranging from *W*_ad_=0.67–0.98 J m^−2^ were predicted for incoherent interfaces of LLZO with BCC Li metal formed from different terminations of both phases (Figure 17a).^[29]^ A reduction of Zr ions at interfacial sites with oxygen coordination less than 6 was observed, particularly for Zr‐rich LLZO surface terminations. In a recent study by Lowe and Siegel, the surface termination of native lithium oxide, Li_2_O, was shown to have a significant impact on the adhesion with Li metal (Figure 17b).^[183]^ Under oxygen rich conditions, the O‐terminated (111) surface of Li_2_O had a work of adhesion that was 30 times stronger with the (111) surface of Li metal than the work of adhesion with the stoichiometric (111) Li_2_O surface, highlighting the importance of oxygen chemical potential on the nature of interfacial adhesion.

In another study, nonstoichiometric Cl terminated (100) surfaces of Li_3_OCl were found to have a much smaller work of adhesion (*W*_ad_=0.09 J m^−2^) with BCC Li metal compared to O terminated surfaces (*W*_ad_=0.75 J m^−2^) (Figure 17c).^[179]^ The Cl‐terminated surface of Li_3_OCl was shown to have a considerably smaller surface energy ($\sigma_{Cl-term}=\;$ 0.19 J m^−2^ at 300 K) than the O‐terminated surface ($\sigma_{O-term}=$ 0.75 J m^−2^ at 300 K). As the (100) surface energy of BCC Li ($\sigma_{Li}=0.45$ J m^−2^) is constant, from Eq. (17), the larger surface energy of the O‐terminated surface resulted in a larger work of adhesion. The strong correlation between the surface energy of the ceramic and the work of adhesion has been highlighted in other studies of metal‐ceramic bonding in both the solid‐state battery and structural engineering fields.[^62^, ^182^, ^198^] The strong correlation between *W*_ad_ and substrate surface energy $\sigma_{sub}$ is also important for understanding the beneficial role of metals and metal alloys as interlayers and current collectors, as metals have intrinsically high surface energies formed from the breaking of metallic bonds, leading to strong adhesion with alkali metals.^[198]^

#### 3.2.4 Interfacial dynamics

The ionic mobility of alkali ions in SSE materials is one of the most important parameters of any SSE material. The diffusivity of alkali ions in bulk SSEs is commonly studied with DFT by one of two approaches: transition state searching methods or ab initio molecular dynamics (AIMD).^[201]^ In AIMD, the forces on atoms within a structure are evaluated with DFT and the positions of the atoms are propagated in time at finite temperature using classical mechanics. The diffusion coefficient can be calculated from the mean square displacement of a collection of the atoms (i. e. Li) over the length of the AIMD run. Due to the large computational cost of DFT calculations, the time lengths that can be simulated with AIMD are typically limited to between 1 ps–1 ns. As ‘rare events’ such as Li hops between sites typically occur on μ s‐ms timescales at ambient temperatures, elevated temperatures are often used to observe diffusion processes.

In transition state searching methods, the activation energies (Δ*E*) for activated processes are calculated at 0 K with DFT. One of the most widely used methods for locating transition states, the climbing image nudged elastic band method (CI‐NEB),^[202]^ allows the position of the transition state in between two known minima structures to be located. The activation energy Δ*E* calculated at 0 K can then be related to the hopping rate, *k*(*T*), and diffusivity *D*(*T*), at finite temperature, *T*, using transition state theory. The long‐timescale dynamics of a system can further be modeled using kinetic Monte Carlo (KMC) methods in which DFT calculated activation energies are used to specify the transition rates between states.^[203]^ Although the majority of computational studies on the dynamics of SSE materials have focused on bulk materials, both transition state searching and AIMD methods have been used in recent years to understand the transport and chemical reactions at the metal anode/SSE interface as discussed below.

##### 3.2.4.1 Interfacial charge transfer

**Figure 18:**
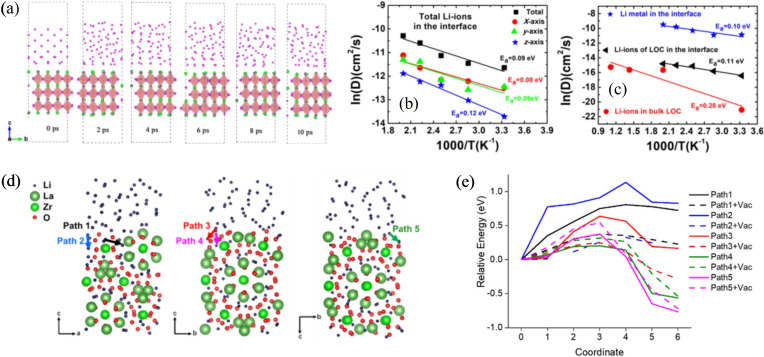
(a) Snapshots from 10 ps ab initio molecular dynamics simulations of Li metal/Li_3_OCl interface. Comparison of diffusivity (*D*) against temperature (*T*) for (b) Li atoms along the *x*, *y*, and *z* directions relative to the Li/Li_3_OCl interface and (c) Li atoms in the bulk Li metal, bulk Li_3_OCl and interface. Activation energies (*E*_a_) are calculated from the gradient of the line. Reprinted with permission from Ref. [204]. Copyright 2020 Elsevier. (d) Diffusion pathways and corresponding activation barriers (e) of Li at the Li metal/LLZO interface predicted with NEB calculations. Adapted from Ref. [205]. Copyright 2019 Wiley‐VCH.

Wu et al. used AIMD calculations to understand the transport of Li at and incoherent Li metal/Li_3_OCl interface (Figure 18a). They showed that the activation energy for Li ion hops along the Li metal/Li_3_OCl interface (0.09 eV) was slightly lower than the activation energy for hops perpendicular to the interface (0.12 eV) (Figure 18b) and considerably lower than in bulk Li_3_OCl (0.28 eV) (Figure 18c), suggesting that the interfacial region of Li/Li_3_OCl may act as a fast pathway for Li ion conduction.^[204]^ The fast transport of Li along the Li/Li_3_OCl interface is consistent with the low work of adhesion (0.119 J m^−2^) for Cl terminated Li_3_OCl surfaces in which Li atoms are weakly bonded. The relationship between the work of adhesion and the interfacial diffusion barriers parallel to the interface is still an area that needs to be explored in these systems.

The activation barrier for Li transport across the Li metal /LLZO interface was calculated by Gao et al.^[205]^ using a combination of NEB and AIMD calculations (Figure 18d) on incoherent Li metal/LLZO interfaces. A low activation barrier of 0.37 eV was predicted for Li transfer across the Li metal/LLZO garnet interface, which is consistent with the very low areal specific resistance (ca. 10^−1^ Ω cm^2^) measured experimentally on clean garnet surfaces.^[109]^ The low charge transfer resistance predicted for these materials strongly supports the conclusion that charge transfer across the Li metal/LLZO interface is not the dominant factor that leads to failure of solid‐state Li metal‐LLZO, and instead other mechanisms such as void formation (see Section 2.2.2) play a more significant role.

Wang et al. recently used AIMD simulations in combination with moment‐tensor machine learning potentials (MTP) to study the interfacial transport across the interface between Li metal and the decomposition products of SSE argyrodite Li_6_PS_5_Cl: Li_2_S, Li_3_P and LiCl.^[206]^ In this study, computationally inexpensive MTPs were trained using AIMD simulations on small (≤ 216 atoms) system sizes and then utilized in MTP‐MD simulations on more realistic Li‐SSE interfaces containing 1000s of atoms. The Li/Li_2_S and Li/LiCl interfaces were found to be considerably more resistive to Li transport that the Li/Li_3_P interfaces, suggesting that the former two decomposition products of Li_6_PS_5_Cl are more detrimental to battery performance.^[206]^

##### 3.2.4.2 Alkali metal void formation

**Figure 19:**
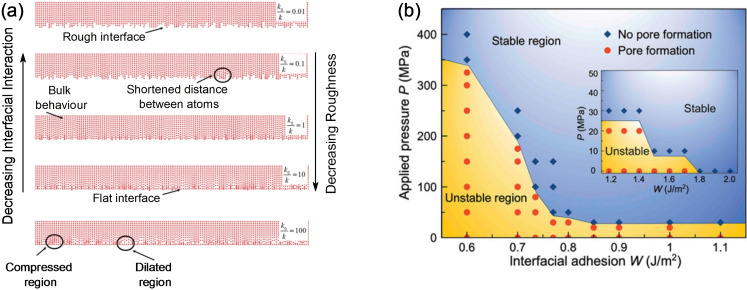
(a) Morphology of a 2D Li metal layer on a model SSE substrate calculated from kinetic Monte Carlo simulations. The energetics of the system are described through the metal‐SSE (*k*_0_) and metal‐metal (*k*) interaction strengths. As the *k*_0_/*k* ratio increases, the interfacial roughness decreases as there is a preference for Li atoms to bind to the SSE. Adapted with permission from Ref. [50]. Copyright 2021 American Chemical Society. (b) Stability of Li metal/SSE interface on Li metal stripping as a function of applied pressure and interfacial adhesion, from large scale molecular dynamics simulations. Pore formation (red dots) was observed leading to an unstable region (yellow) for interfaces with low interfacial adhesion unless high pressures are applied. Stable (blue) pore free stripping was only achieved with zero applied pressure for interfacial adhesion values >1.8 J m^−2^. Adapted from Ref. [111]. Copyright 2021 Wiley‐VCH.

The morphology of Li metal deposition at a model Li metal/SSE interface was studied by Tewari and Mukherjee using KMC simulations (Figure 19a). A simple harmonic potential was used to describe the energetics of Li‐Li and Li‐substrate interactions.^[50]^ When the strength of the Li‐substrate interactions was considerably stronger than the Li‐Li interactions (i. e., a strong work of adhesion), a flat Li metal/SSE interface was observed. When the Li‐substrate interactions were smaller than the Li‐Li interactions (i. e., a low work of adhesion), significant roughening of the interface occurred during electrodeposition, highlighting the direct link between interfacial bonding and Li metal surface structure.

Large scale classical molecular dynamics simulations coupled with AIMD calculations were recently used to understand how the work of adhesion and applied pressure affect void formation at model Li metal/SSE interfaces (Figure 19b).^[111]^ It was demonstrated that when the work of adhesion been Li metal and the SSE was less than 0.6 J m^−2^, external pressures of hundreds of MPa were required to suppress void formation. Works of adhesion of >1.8 J m^−2^ were required to suppress void formation without the application of significant pressures. Interfacial coherence was also shown to play an important role in the nature of defects at the Li metal/SSE interface through large scale dynamics models. At coherent BCC Li metal/SSE interfaces, diffusion of Li vacancies from the interface into the bulk during stripping was found to be rapid, helping to suppress void formation. For semicoherent and incoherent interfaces with larger lattice mismatch, the BCC Li metal structure became disordered at the interface.^[207]^ The disordered Li layer absorbs Li vacancies and reduces the amount of mobile Li vacancies that can move into the bulk, promoting void formation. The role of interfacial adhesion and electronic structure on void formation was further studied by Yang and Qi, using DFT coupled with KMC. Vacancy accumulation was suppressed at a Li(001)/Li_2_O(110) interface compared to a Li(001)/LiF(001), due to the formation of stronger interfacial Li‐O bonds in the former system.^[208]^

**Figure 20:**
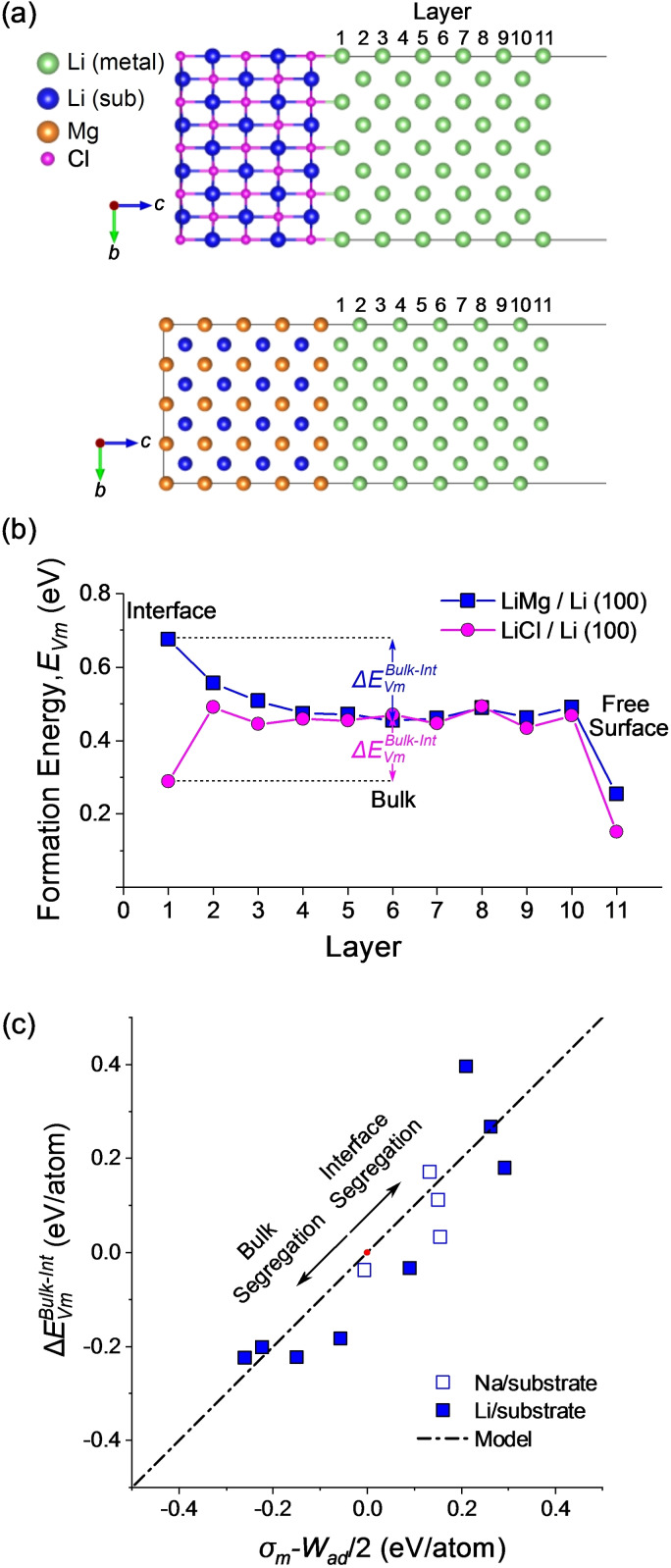
(a) Nonreactive, single coherent interface models between the (100) surface of BCC Li metal and the (100) surfaces of LiCl and LiMg. (b) Formation energy of Li vacancies as a function of distance from the interface. A positive segregation energy (Δ$E_{Vm}^{Bulk-Int}$ ) was observed for LiCl, whereas a negative segregation energy was observed for LiMg (Δ$E_{Vm}^{Bulk-Int})$ . Plot of segregation energy (Δ$E_{Vm}^{Bulk-Int}$ ) with alkali metal surface energy ($\sigma_{m}$ ) and interfacial work of adhesion (*W*_ad_) for a series of oxide, halide, phosphate, and metallic alloy substrates. The universal relationship in Eq. (20) is shown as a dot‐dash line. Segregation of vacancies to the alkali metal/substrate interface is predicted to occur when $\sigma_{m}-\frac{W_{ad}}{2}>0$ . Adapted with permission from Ref. [198]. Copyright 2021 Royal Society of Chemistry.

In a recent work by some of the current authors, a simple bond‐breaking model coupled with explicit first principles interface calculations was developed to understand the relationship between the interfacial work of adhesion (*W*_ad_), alkali‐metal surface energy ($\sigma_{m}$ ) and segregation energy (Δ$E_{Vm}^{Bulk-Int})$ for a Li (Na) vacancy to move from the alkali metal/SSE interface into the bulk of the alkali metal (Figure 20a and 20b).^[198]^ This work built upon the previous study by Kumar et al.^[2]^ which investigated Ni vacancy formation and diffusion at coherent Ni/α ‐Al_2_O_3_ interfaces. A universal relationship was proposed in which the vacancy segregation energy was given by Eq. 20:

(20) $$\Delta E_{Vm}^{Bulk-Int}=\frac{\sigma_{m}-\frac{W_{ad}}{2}}{\rho }$$

Where ρ is the surface density of Li atoms.^[198]^ This relationship was found to hold for a wide range of nonreactive oxides, halides and metals using explicit DFT calculations (Figure 20c). A negative value of Δ$E_{Vm}^{Bulk-Int}$ results in a preference for metal vacancies to be injected into the bulk away from the interface, suppressing void formation. From Eq. (20), negative values of Δ$E_{Vm}^{Bulk-Int}$ occur when $W_{ad}$ >2$\sigma_{m}$ . Substituting the Young–Dupré equation [Eq. (5)] into this relationship shows that a contact angle of $\theta =0^{\circ }$ , i. e. perfect wetting, is required to give a negative Δ$E_{Vm}^{Bulk-Int}$ value and suppress void formation. The model was further extended to treat the incoherent Li (100)/LiCl (100) interface in which it was found that the vacancy segregation energy Δ$E_{Vm}^{Bulk-Int}$ was very sensitive to the bonding environment of Li metal atoms at the interface. The average $W_{ad}$ value calculated for an incoherent interface was therefore not a sufficient metric to predict the void formation behavior and instead the vacancy segregation energy Δ$E_{Vm}^{Bulk-Int}$ of the most weakly bound Li metal surface site was proposed as a better descriptor of void formation.

An important result from this work was that the condition of $W_{ad}$ >2$\sigma_{m}\;\theta =0^{\circ }$ in Eq. (20) is not met by many nonreactive Li/SSE interfaces or interfaces between Li and decomposition products such as Li_3_P, LiCl and Li_2_S in commonly studied SSE systems, such as LLZO or Li_6_PS_5_Cl.^[198]^ The poor interfacial adhesion between different decomposition products and Li and Na metal is further supported by a recent large scale computational screening work by Wang et al., in which they showed that only certain high energy surfaces of Li_2_O, Li_3_N and Na_2_O meet the $W_{ad}$ >2$\sigma_{m}$ criteria, where‐as the majority do not.^[206]^

The universal relationship in Eq. (20) also highlights an important result that metallic alloys such as Li_1‐x_Mg_x_ are particularly promising candidates as interlayers at the alkali metal/SSE interface, as they have intrinsically high surface energies which lead to large values of $W_{ad}$ with alkali metals.^[198]^ The experimental development of metallic interlayers and alloys will be discussed further in Section 4.3.

#### 3.2.5 Multiscale models

DFT calculations provide detailed quantum mechanical information about the atomistic structure at the alkali metal/SSE interface, but due to the limited system size (<1000 atoms) that can be routinely studied, multiscale models are required to understand the evolution of the microstructure on larger length scales. One of the most powerful family of multiscale techniques that have been developed in recent years for modeling the evolution of interfaces in rechargeable batteries at the particle level are phase field models.[^209^, ^210^] Phase field models treat the phase boundary between two materials or ‘phases’ as a diffuse interface described by phase field variables, φ . The phase field variables are dictated by partial differential equations that are governed by the transport equations of the materials under study. Phase field models have been particularly successful in understanding the formation of Li dendrite structures in liquid electrolyte systems as a function of parameters such as the applied voltage^[211]^ and electrolyte mass‐transfer.^[212]^ A powerful phase field model was recently developed by Zhao, Wang and Martínez‐Pañeda to study void formation at the Li metal/SSE interface which could capture both creep of the Li metal under applied pressure in addition to the diffusion and creation/annihilation of Li vacancies.^[213]^

**Figure 21:**
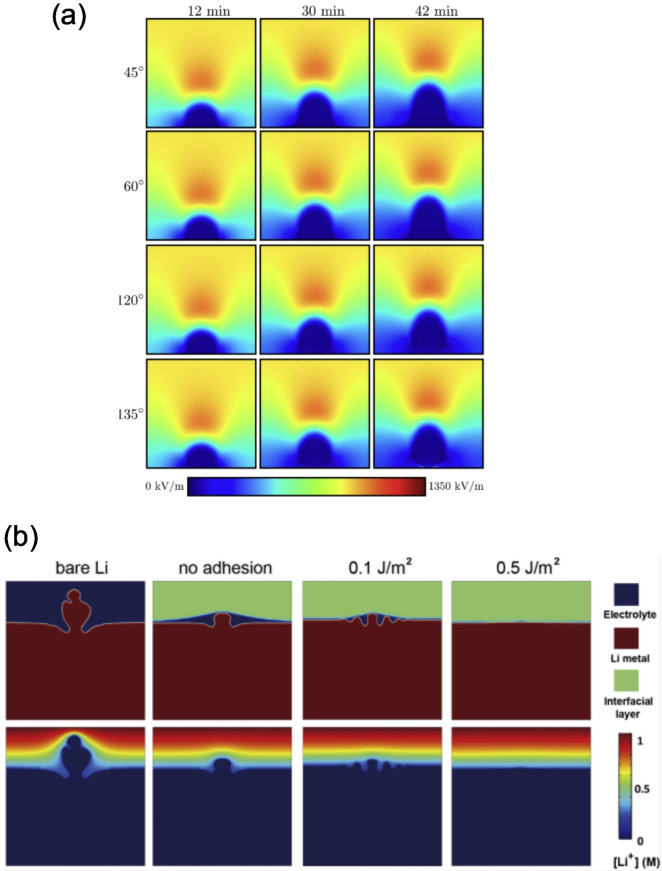
(a) Phase field model of time evolution of Li deposit morphology as a function of substrate wetting angle. The colors show the electric field distribution at the Li deposit. Reproduced with permission from Ref. [215]. Copyright 2014 Elsevier. (b) Phase field model of Li dendrite growth under LiAl interlayer as a function of the work of adhesion. A smooth Li deposition is only observed when the work of adhesion reached 0.5 J m^−2^. Adapted with permission from Ref. [217]. Copyright 2019 Elsevier.

However only a handful of studies have directly studied how the strength of the wetting between the alkali metal and other solid components, such as the SSE, solid interlayers or the current collector affect the growth morphology and kinetics. Ely et al. investigated how the work of adhesion/contact angle of a Li deposit on a substrate influenced the growth kinetics.[^214^, ^215^] It was demonstrated that substrates with small wetting angles (large adhesion strengths) strongly modified the shape of Li deposits from spherical to lenticular (Figure 21a). Stronger adhesion between the substrate and Li deposit leads to enhanced lateral growth which favors thin film growth. Weak adhesion leads to the kinetic instabilities of the Li deposit that can lead to detachment at high current densities. This model was further extended to interaction between metallic Li dendrites and another solid component of liquid electrolyte batteries, the separator, in which it was shown that higher interfacial adhesion led to more uniform Li growth.^[216]^

Phase field modeling was recently used to investigate the role of solid interlayers in suppressing Li dendrite growth in liquid electrolyte cells.^[217]^ When the work of adhesion between Li and the interlayer was low (<0.1 J m^−2^) inhomogeneous dendritic structures were observed underneath the coating layer (Figure 21b). When the work of adhesion reached 0.5 J m^−2^, a flat, smooth surface of Li was observed underneath the coating. As the surface energy of Li used in this study was 0.439 J m^−2^, a work of adhesion of adhesion of 0.5 J m^−2^ corresponds to a wetting angle of θ= 80.6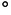 from Eq. (5). Based on the design principle of maximizing wetting between the interlayer and Li metal, they studied a nanostructure LiAl alloy as an interlayer, formed from the decomposition of AlCl_3_.^[217]^ DFT calculations predict a strong work of adhesion between Li and LiAl of 1.54 J m^−2^ (${\theta \approx 0)}^{\circ }$ due to the metallic nature of bonding between both phases. Improved cycling performance was observed for the Li/LiAl system compared to bare Li, with stable plating and striping observed at 5 mA cm^−2^ for over 500 h.

The development of phase field models that include the interfacial work of adhesion on model flat interfaces has been an important step for understanding the morphological evolution of alkali metal/SSE interfaces. However, further development of phase field and other multiscale models is urgently required to understand the impact of more complex factors such as surface roughness and inhomogeneity on interfacial adhesion that occur in real systems.

## 4 Experimental Strategies to Control Wetting

This section reviews the development of materials engineering strategies to improve interfacial adhesion in alkali metal SSBs. Particular focus is directed towards analyzing the fundamental processes that lead to improved adhesion in each case, using the scientific framework developed in the previous sections.

### 4.1 Liquid metal/SSE interfaces: past and present

To date, alkali metal batteries with solid electrolytes have only reached technological maturity and commercial development in two high temperature (around 350 °C) applications: NaS and ZEBRA batteries.[^218^, ^219^, ^220^, ^221^, ^222^] The acronym BASE batteries, often employed to group the families of NaS and ZEBRA batteries, stands for “Beta Alumina Solid Electrolyte” which is the most frequent ceramic separator used in these batteries. BASE batteries operate above the melting point of Na and interfacial wetting in such systems therefore draws on the concepts of liquid/solid wetting. Some instructive parallels can be made between the strategies developed to improve wetting at liquid/solid interfaces and solid/solid interfaces.^[223]^

**Figure 22:**
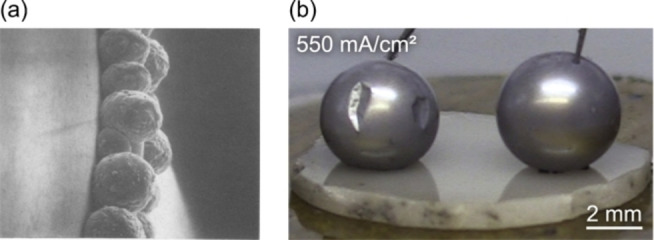
(a) SEM micrograph showing the intrinsically poor wetting of a Na/Na‐β ’’‐alumina interface at 230 °C (60X magnification). Reprinted with permission from Ref. [224]. Copyright 1983 North‐Holland Publishing Company. (b) Synchronized electrochemical testing and optical observation of molten Li on LLZO (the distorted shape of the left droplet is associated to a thin Li_2_O surface passivation shell). Adapted with permission from Ref. [116]. Copyright 2021 Elsevier.

Early in the development of the NaS battery, improving wetting at the molten Na/Na‐β’’‐Al_2_O_3_ interface (Figure 22a) was identified as the key for preventing degradation and improving cell performance.[^94^, ^224^] Reacting with atmospheric moisture, the surface of Na‐β/β’’‐Al_2_O_3_ can get covered by a Na_2_O layer, which increases the interface resistance and impedes efficient interfacial charge transfer at the Na metal electrode[^94^, ^223^, ^225^] Several studies also mention the problem of Ca impurities contained in the Na metal electrode or at the Na‐β/β’’‐Al_2_O_3_ interface which easily get oxidized to form a CaO layer on the Na‐β/β’’‐Al_2_O_3_ surface.[^226^, ^227^] The adhesion of Na with CaO is intrinsically poor due to the ionic nature of wide band gap insulator CaO, as previously discussed in Section 2.1.4.2.

Two strategies relying on reactive wetting have been widely employed to combat the poor wettability of Na metal on Na‐β/β’’‐Al_2_O_3_: (1) the Na‐β/β’’‐Al_2_O_3_ surface can be coated with an alloying metallic layer (commonly Pb or Bi) during manufacturing;^[228]^ (2) Al or Ti, in the form of foam or powder, can be mixed in the molten Na metal to react with Na_2_O present at the Na‐β/β’’‐Al_2_O_3_ surface and form ternary phases (Na_x_M_y_O_z_, where M=Al or Ti).[^222^, ^228^] The combination of both strategies yielded the best results. The improved interface adhesion was demonstrated by a reduction of the cell resistance during cycling.

Operating SSBs above the melting point of alkali metals (around 180 °C for Li and 98 °C for Na) suppresses the morphological instabilities characteristic of solid/solid interfaces as was recently demonstrated by the staggering cycling rates of molten Na/Na‐β’’‐Al_2_O_3_ and molten Li/LLZO interfaces (in the range of 10^2^–10^3^ mA cm^−2^).[^12^, ^116^] The step change in diffusivity and viscosity above the melting point of the alkali metal is suggested as the cause for the increase in cycling performance.

### 4.2 Impact of surface composition on experimentally measured wetting

As described in Section 2 of this review, wetting is governed by the surface chemistry of the two interfacing materials. Materials engineering strategies to improve adhesion at an alkali metal/solid electrolyte interface therefore require an intricate understanding of the surface composition of both phases.

Surface compositions may vary from the bulk material as a result of how the SSE was processed and handled prior to being used in a cell. Experimental conditions that influence surface chemistry can include both the thermal history of the sample (which can affect the segregation of mobile dopants, promote the nucleation of secondary phases, or favor the formation of complexions to minimize surface energies) or how the sample was processed after sintering (for instance which atmosphere it was handled in). The stabilized surface chemistry can either have detrimental or beneficial effects on wetting.

**Figure 23:**
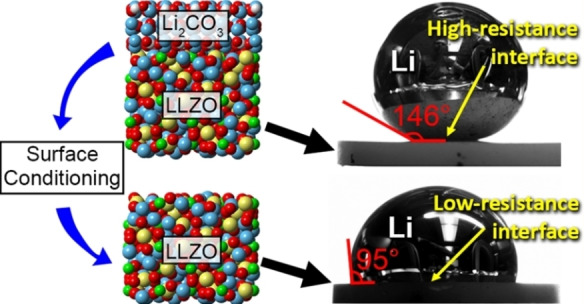
Impact of wettability of a liquid Li metal droplet on the surface of LLZO with (top) and without (bottom) a Li_2_CO_3_ contaminant layer. Contact angle θ is shown in red. Reproduced with permission from Ref. [43]. Copyright 2017 American Chemical Society.

An example of the detrimental impact that surface chemistry can have on wetting is the notorious case of contaminants on the surface of air sensitive SSEs. The reactivity of LLZO has been widely studied and the presence of Li_2_CO_3_ and LiOH on its surface are now collegially attributed to a two step reaction with atmospheric H_2_O and CO_2_ when samples are handled, even briefly, in air.[^79^, ^80^, ^229^, ^230^] The lower work of adhesion between Li metal and Li_2_CO_3_ (evaluated by DFT calculations to be between 0.1 and 0.26 J m^−2^)[^43^, ^76^] in comparison to bulk LLZO and Li metal (between 0.67 and 0.98 J m^−2^)[^29^, ^43^] explains the large Li/LLZO interface resistance and low tolerance to Li filament (dendrites) penetration in air exposed LLZO (Figure 23). Preventing the formation of Li_2_CO_3_ and LiOH on LLZO is possible if critical steps of the synthesis are carefully controlled in a protective atmosphere.[^23^, ^33^, ^80^]

Alternatively, if preventing contamination is impossible, LiOH and Li_2_CO_3_ can be removed by polishing, followed by an optional post‐polish annealing step[^11^, ^23^, ^43^, ^110^] or by acid etching.^[231]^ The in situ formation of a moisture protective passivating film by adding LiF to LLZO is another possible route to prevent contamination.^[232]^ When contaminants are absent from the LLZO surface (for instance by forming the Li/LLZO interface in situ under the high vacuum enviroment of a SEM chamber), interface resistances as low as 1 Ω cm^2^ can be obtained.^[109]^ The Na metal/Na‐β/β’’‐Al_2_O_3_ interface is also strongly affected by surface contaminants.^[11]^ In a recent study, Bay et al. demonstrated that the nature of surface contaminants on Na‐β/β’’‐Al_2_O_3_ is not limited to Na_2_O (as concluded from the studies cited in the previous section[^94^, ^223^, ^233^]) but also includes hydroxide and carbonate species. The removal of surface contaminants was suggested as the main cause for the interface resistance improvement from 19.9 kΩ cm^2^ to 8 Ω cm^2^.

Contaminants on the surface of akali metal foils should also be considered carefully. It is important to note that a concentration of 0.1 ppm (which is the typical target value for O_2_ and H_2_O in gloveboxes) corresponds to a partial pressure of 10^−4^ mbar and to an impegement rate of around 3x10^16^ cm^−2^ s^−1^ (for O_2_ at 25 °C). Thus, a typical surface with around 10^15^ atoms.cm^−2^ like Li metal can form a monolayer of Li_2_O in only a few seconds even at a glovebox concentration of 0.1 ppm. The formation of oxides on the surface of Li metal handled in a glovebox has been demonstrated experimentally in several studies.[^136^, ^234^, ^235^] Even in ultra high vacuum instruments, alkali metals can form a passivation layer in a few hours. The presence of a Li_2_O film forming rapidly on Li metal droplets inside a glovebox was suggested as a source of spreading resistance on coated substrates.^[75]^ As discussed in Section 2.1.6, the existence of spreading resistance can be detected with dynamic contact angle measurements by looking for a large hysteresis between the advancing and receding contact angles.^[86]^

Although uncommon, adventitious species forming on some SSEs can improve their wettability towards the metal anode. One example is the surface engineering of Na_3_SbS_4_ electrolytes. Upon exposure to atmospheric moisture for a few minutes, Tian et al. demonstrated that a hydrated Na_3_SbS_4_.8H_2_O surface forms with a better adhesion and stability towards Na metal than the dry Na_3_SbS_4_ electrolyte.^[236]^ The improved initial wetting can be attributed to a reactive wetting mechanism whereby Na reacts with the hydrated surface to form NaH and Na_2_O. It remains to be demonstrated whether these decomposition species have a good work of adhesion against Na metal and will prevent void formation at high current densities.

When surface species (such as Li_2_CO_3_ on LLZO or hydroxide species on Na‐β/β’’‐Al_2_O_3_) are detrimental to adhesion, their removal is a necessary condition to achieve better wetting. Yet, the work of adhesion between a contaminant‐free SSE and alkali metal might still not be high enough to prevent interfacial pore formation during stripping in the absence of large applied pressures.^[111]^ For example, the contact angle measured by Sharafi et al. in their seminal paper regarding the impact of Li_2_CO_3_ on the wettability of LLZO by Li metal (Figure 23) was estimated to be 95°. By definition, this cannot be considered as good wetting and it is only good relative to the non‐annealed LLZO surface which displays an extremely poor wettability. As discussed in Section 3.2.4.2, the prevention of void formation requires contact angles approaching 0°.

In the following section, materials engineering strategies that enhance the interfacial adhesion with alkali metals are discussed.

### 4.3 Interfacial engineering with interlayers

Maintaining the morphological integrity of the alkali metal/SSE interface during stripping and plating cycles is crucial to guarantee the performance of SSBs over numerous charge/discharge cycles. Whilst interfacial contact can be constrained by externally applied pressures, the commercial applicability of such a strategy is debated (especially if the pressures involved are in the order of several hundreds of MPa). A widely adopted strategy to improve adhesion at the alkali metal/SSE interface consists of employing interlayers with high wettability towards alkali metals.

Interlayers are typically coated on the SSE surface via a thin‐film deposition technique. A variety of interlayer chemistries (metal, metal oxide, polymer, carbonaceous, etc.) with improved wettability towards alkali metals have been identified in experimental studies. In most cases, the mechanism by which interlayers improve adhesion involves reactive wetting (Section 2.1.4).

Interlayers are also often investigated as buffer layers to protect the surface of SSE which are not electrochemically stable against alkali metals. These two use cases of interlayers (wetting and protection) are often separated in the literature, but the wettability of protective interlayers should be added to the selection criteria list to ensure that the gain in electrochemical stability will not result in void formation during cycling. Reaching thermodynamical equilibrium to prevent the decomposition of the SSE whilst maintaining good wetting is an additional consideration in this case. A challenge with interlayer‐based strategies is to ensure a good adhesion of the interlayer to the SSE surface and to prevent the dissolution of the interlayer into the bulk of the electrode over the lifetime of the cell.

#### 4.3.1 Metal and metal oxide interlayers

##### 4.3.1.1 Reactive wetting

**Figure 24:**
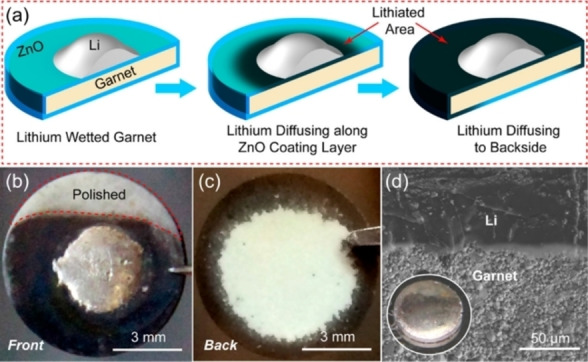
An example of an interlayer with fast reaction kinetics, ZnO on LLZO: (a) Schematic depiction of Li spreading on the ZnO interlayer. (b) Top side and (c) back side of the ZnO‐coated LLZO pellet in contact with Li metal. (d) Cross‐section SEM image of the Li/ZnO/LLZO interface. Reproduced with permission from Ref. [247]. Copyright 2017 American Chemical Society.

Examples of wetting induced by metal and metal oxide interlayers are abundant in recent literature. In most cases, the interfacial contact is improved via a reactive wetting mechanism. The multitude of Li‐X or Na‐X dual alloys (not even including tri elements alloys) crossed with the multitude of SSEs has spurred a plethora of experimental studies. A nonexhaustive list of Li‐X systems used as interlayers include: Li‐Au,^[237]^ Li‐Ge,^[238]^ Li‐Sn,[^239^, ^240^] Li‐Si,[^239^, ^241^] Li‐Zn,^[239]^ Li‐Mg^[242]^ and Li‐Ag.[^243^, ^244^] Li‐Ag alloys have been used, for example, with Li_6_PS_5_Cl electrolytes to stabilize the interface through the formation of two alloy compositions and a mixed interlayer between the anode and electrolyte which prevents their reaction.^[245]^ Three‐element alloys such as Li‐Cu‐Sn^[240]^ have also been reported. Ga_2_O_3_ ^[229]^ and In_2(1‐x)_Sn_2x_O_3_ (ITO)^[246]^ are other examples of Li‐X‐O systems which have been reported to improve the wettability of LLZO. Significant improvements in Li metal wettability were also obtained with ZnO interlayers, whether on an inert substrate,^[75]^ on LLZO[^247^, ^248^] or on LATP.^[249]^ For interlayers with fast reaction kinetics against alkali metals, a minimization of the contact angle (spreading) can typically be noticed visually if a droplet of liquid metal is deposited on the interlayer surface. Figure 24 shows the reactive wetting that Wang et al. were able to observe when they pressed a piece of solid Li metal on a LLZO surface coated with a ZnO interlayer.^[247]^

Owing to the popularity of LLZO as a SSE, the Li/LLZO interface has gained the most interest. Among all metal or binary oxide interlayers used to improve the wettability of LLZO, Al^[158]^ and Al_2_O_3_ ^[76]^ interlayers are probably the most emblematic. The obtention of a negligibly low interfacial resistance (2 Ω cm^2^) at the Li/LLZO interface using an Al_2_O_3_ interlayer deposited by atomic layer deposition (ALD) by Han et al. has been a key achievement putting interlayers to the forefront. Using DFT calculations, the group demonstrated that the Al_2_O_3_ interlayer is lithiated to form a new Li_x_Al_2_O_3+x/2_ phase (x=0.4 to 1.4) with higher wettability towards Li metal.^[76]^ In another DFT study, Li_9_Al_4_ and Li_2_O were determined to be the equilibrating phases of the Li‐Al‐O system against Li metal, with a reaction energy for lithiation of −0.23 eV/Li.^[250]^

Whilst the efforts required to prevent or remove the presence of Li_2_CO_3_ on the surface of LLZO could be considered as a barrier for industrial applications, a recent study from Meng et al. introduced a simplified route to mitigate the negative influence of Li_2_CO_3_ on LLZO.^[229]^ The strategy relies on the in situ formation of a lithiophilic Ga_2_O_3_ interlayer by directly painting liquid metal Ga on the surface of a passivated LLZO. Liquid metal Ga was used to break the Li_2_CO_3_ layer on the surface of LLZO and form a lithiophilic Ga_2_O_3_ skin. Unlike studies mentioned in the previous section, a clear advantage of this strategy is that LLZO can be processed freely in air while maintaining an ultimate Li/LLZO interface resistance below 5 Ω cm^2^. The excellent electrochemical performance obtained with this strategy is also illustrated with the high critical current density to dendrite formation (1.7 mA cm^−2^) and the ability of the SSE to be cycled for hundred of hours at 1 mA cm^−2^. An interesting parallel can be made with the Pb coating strategy which was widely adopted to mitigate the detrimental effect of contaminants on the surface of Na‐β/β’’‐Al_2_O_3_ in liquid Na BASE batteries (see Section 4.1).^[223]^

For Na systems, the majority of studies involving interlayers were conducted on Na‐β/β’’‐Al_2_O_3_. A minor improvement in the wettability of Na‐β/β’’‐Al_2_O_3_ by Na metal was obtained by using a Bi interlayer.^[251]^ A more significant improvement was obtained by coating a Sn layer on a contaminant‐free Na‐β/β’’‐Al_2_O_3_ surface.^[59]^ These last results highlight a point already stressed in the previous section: the removal or breaking of surface contaminants is necessary but not sufficient to significantly improve the wettability of Na‐β/β’’‐Al_2_O_3_; only the addition of a sodiophilic interlayer enabled contact angles to approach 0°.

##### 4.3.1.2 Kinetically limited reactive wetting

Not all metallic interlayers have as fast reaction kinetics as the examples presented in the previous section. For interlayers with slower reaction kinetics, the wetting of the interlayer is achieved during the first cycles of plating and stripping. A good example was provided by Yang et al. who investigated TiO_2_ as an interlayer to improve the wettability of NZSP towards Na metal.^[252]^ They found that TiO_2_ is a kinetically limited reactive interlayer which reacts with Na to form a Na_x_TiO_2_ phase after a few activation cycles. They identified that interfacial contact was improving in the first cycles of plating and stripping through a decrease and stabilization of the Na/TiO_2_‐NZSP interface resistance. Cells employing the TiO_2_ interlayer displayed a higher tolerance to extended cycling and void formation.

##### 4.3.1.3 Nonreactive wetting

To date, most interlayers employed to improve the wettability of SSEs have relied on a reactive wetting mechanism. However, as discussed in Section 2.1.4, wetting can also be obtained between two nonreacting phases. In particular, metals should in theory always wet other metals well. Therefore, employing metallic interlayers which do not react with alkali metal is another possible route to improve the wettability of SSEs.

A nonreactive interlayer was recently proposed as a solution to prevent void formation at the Li/LLZO interface. In this study, Raj et al. compared the behavior of a nonreactive W interlayer with that of a reactive Al interlayer.^[253]^ They employed first principle calculations to construct the Wulff shapes of Al and W and demonstrated that W facets have a higher surface energy than Al ones which, according to Eq. (4), should result in a lower work of adhesion for the Al interlayer. The more favorable formation of voids on the low energy Al facets in comparison to the W ones was experimentally demonstrated by cycling tests where an overpotential associated with void formation was detected at lower current density for the cell with an Al interlayer than for the W one.^[253]^

##### 4.3.1.4 Surface decoration

In most cases, researchers employ interlayers which fully cover the SSE surface. However, a few studies have demonstrated that surface decoration with smaller islands can also promote spreading via reactive wetting. Such a strategy was employed by Jin et al. who decorated the surface of Na‐β/β’’‐Al_2_O_3_ pellets with Bi or Pb islands.[^254^, ^255^] In the first study, Bi islands were found to result in better wetting at the Na/Na‐β/β’’‐Al_2_O_3_ interface.^[254]^ In the second study, the wetting mode of Pb‐decorated Na‐β/β’’‐Al_2_O_3_ was described as Wenzel‐type, evolving to a sunny‐side drop with the spreading of Na.^[255]^ The impact of surface topography on wetting is explored in more details in Section 4.5.

#### 4.3.2 Polymer interlayers

Using polymers to improve the stability of metal anodes is a research path reported in the fields of both SSBs and liquid electrolyte batteries. Whilst the polymer interlayer is coated on the metal surface for liquid electrolyte batteries, it is more common to coat it on the SSE in the case of SSBs. For lithium‐ion batteries, polymer interlayers typically act as engineered solid‐electrolyte interfaces (SEIs) helping to homogenize Li plating.^[256]^ Uniform Li plating is essential to prevent continuous SEI formation (and its associated problem of low Coulombic efficiency) and dendrite propagation. Ensuring good adhesion between Li metal and the polymer interlayer is therefore a central goal. In a study using a copolymer interlayer, Wang et al. suggested a reactive wetting mechanism to explain the strong adhesion between the interlayer and Li metal.^[256]^ The copolymer interlayer successfully prevented dendrite formation and achieved good performance at high current densities (1000 cycles at 5 mA cm^−2^ /10 mAh cm^−2^).

Polymer interlayers are also starting to be used to improve the alkali metal/SSE interface in SSBs. Within the larger family of hybrid electrolytes, the ones employing polymer electrolyte interlayers are called layered (or sandwich) structures. A detailed review focusing on the ionic transfer kinetics at such heterogeneous interfaces was recently published and summarizes recent developments in the field.^[257]^ The remainder of this section will focus on layered structures and the impact on wetting of polymer electrolytes used as interlayers.

In such architectures, the SSE is used as a separator preventing crosstalk between the electrodes and providing a barrier against dendrites. The polymer interlayer provides good ductility which enables good contact between the metallic anode and SSE, despite large volume changes, and it homogenizes the charge carrier flux, leading to a more stable plating and stripping. Often, the primary role of the polymer electrolyte interlayer is not as a contact mediator, but rather as a buffer between two phases which would otherwise react to form detrimental by‐products. For this reason, polymer electrolyte interlayers have been particularly studied in combination with reactive SSE such as LAGP,[^258^, ^259^, ^260^] LATP,^[261]^ LGPS,^[262]^ Li_6_PS_5_Cl,^[263]^ Li_10_SnP_2_S_12_,^[264]^ or Na_3_SbS_4_.^[265]^ Polymer interlayers have also been studied on less reactive SSE such as LLZO[^232^, ^266^, ^267^, ^268^, ^269^] or NZSP.[^270^, ^271^] For air sensitive SSE such as LLZO, the same precautions need to be observed regarding the removal of surface contaminants prior to coating with the polymer electrolyte interlayer to minimize the interface resistance.^[266]^ Because of its high ionic conductivity in comparison to other polymers, poly(ethylene oxide) (PEO) mixed with various ion conducting salts (LiTFSI, LiTf, LiClO_4_, etc.) has been used in the majority of publications regardless of the nature of the SSE.[^232^, ^258^, ^263^, ^264^, ^265^, ^266^, ^267^] A good illustration of the effectiveness of polymer interlayers was provided by Fu et al. who demonstrated that a symmetric Li/PEO/LLZO/PEO/Li cell could be cycled at the high current density of 1 mA cm^−2^ without significant polarization or short‐circuits.^[269]^ The 2 μm PEO interlayer they used resulted in more uniform plating and stripping of Li^+^ ions preventing the formation of voids and dendrites unlike a noncoated Li/LLZO/Li cell which short‐circuited in less than an hour at a current density of 0.3 mA cm^−2^.

**Figure 25:**
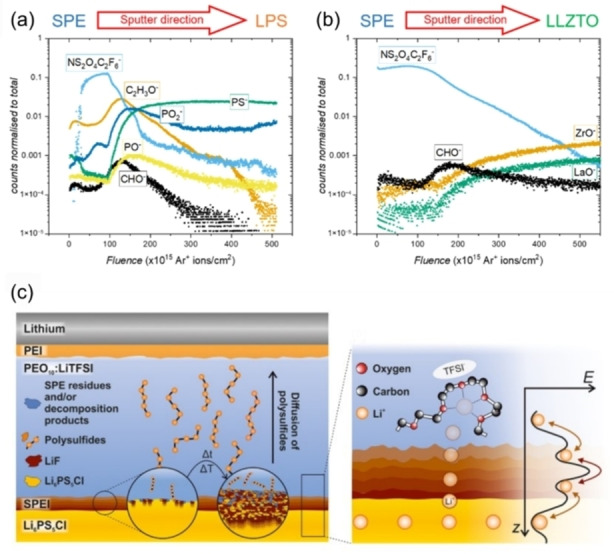
(a) ToF‐SIMS depth profiles across a PEO:LiTFSI/Li_3_PS_4_ interface showing the presence of an interphase. (b) ToF‐SIMS depth profiles across a PEO:LiTFSI/LLZO interface showing less mixing of the phases (SPE: solid polymer electrolyte). Reproduced with permission from Ref. [273]. Copyright 2022 by authors (CC‐BY license). (c) Schematic depiction of the decomposition reactions occurring at the interfaces between a PEO:LiTFSI interlayer, Li metal, and Li_6_PS_5_Cl. Reproduced with permission from Ref. [263]. Copyright 2019 American Chemical Society.

Despite showing very promising results, polymer interlayers also come with several issues that need to be more fundamentally understood and resolved. First, an intimate contact between the polymer electrolyte interlayer and the SSE should be achieved as any voids at the interface will increase the tortuosity of ions and inevitably result in an overall increased cell resistance. Researchers have typically used hot pressing to improve the polymer/SSE interfacial contact.[^263^, ^266^, ^272^] But reactive wetting strategies can also be developed. In a recent study, Spencer Jolly et al. compared the interfaces of a polymer electrolyte (PEO with LiTFSI as Li salt) with LLZO and Li_3_PS_4_.^[273]^ Using ToF‐SIMS and depth‐profiled XPS, they demonstrated that the PEO:LiTFSI/Li_3_PS_4_ interface was electrochemically unstable and formed a stabilizing interphase, contrary to the PEO:LiTFSI/LLZO interface which remained stable (Figure 25). Surprisingly, the interface with the lower resistance was the reactive PEO:LiTFSI/Li_3_PS_4_ interface. Although not explicitly mentioned in their study, a possible explanation for this could be that the PEO:LiTFSI/Li_3_PS_4_ interface has an improved contact because of reactive wetting. The same polymer electrolyte (PEO:LiTFSI) has also been reported to react against Li_6_PS_5_Cl^[263]^ (Figure 25) and Li_10_SnP_2_S_12_.^[264]^ In both cases, the formation of a resistive interphase led to an increase of the interface resistance. A balance between the formation of a resistive interphase and an improved contact should therefore be sought.

A lack of experimental information about the intrinsic reactivity and interfacial adhesion between polymers and alkali metals has so far hindered materials design. Future efforts should be directed to careful contact angle measurements between alkali metals and polymer electrolyte interlayer materials, in combination with first principles calculations as highlighted in Section 3.2.2.

#### 4.3.3 Carbonaceous interlayers

Carbonaceous materials are being investigated as ductile interlayers with good electronic conductivity which are believed to promote a more uniform plating.[^248^, ^252^] The morphology of carbon based interlayers is diverse with reports of graphene‐like interlayers,^[274]^ graphitic interlayers,^[275]^ or carbon nanotubes.^[248]^ The use of a graphene‐like interlayer at the Na/NZSP interface resulted in a tenfold reduction in interface resistance to 46 Ω cm^2^ at room temperature, and a great improvement in the cycling stability at high current densities up to 2 mA cm^−2^.^[252]^ In this case, the interlayer had to be grown using CVD at a low enough temperature to ensure the presence of defects in the graphene layer, which were suggested to help fast Na^+^ transport across the interface. Sophisticated deposition techniques are not always required as demonstrated by Shao et al. when they improved wetting at the Li/LLZO interface by drawing a graphite interlayer with a pencil.^[275]^ A simple technique was also reported by Xu who coated carbon nanotubes on a LLZO surface with an infiltration technique.^[248]^ The stabilization of the interface and minimization of its resistance usually occurs over the course of the first few plating/stripping cycles during which lithium is intercalated in the graphite structure to form the lithiated interlayer.^[248]^ To confirm this experimental hypothesis, Shao et al. used DFT calculations to demonstrate the highly favorable enthalpy of formation of lithiated graphite LiC_6_ on contact between Li metal and their graphite coating.^[275]^

#### 4.3.4 Protective interlayers: balancing stability and wettability

The primary function of protective/buffer layers is to prevent the detrimental reduction of SSEs by alkali metals. To be efficient, a buffer layer needs to either be readily thermodynamically stable against alkali metals or react to form decomposition products that are stable. In this regard, a wide range of electronically insulating nitride and oxynitride materials are predicted to be stable against Li metal and could be used as protective buffer layers.^[250]^

The introduction of protective interlayers should not elude the problem of interface wettability. Maintaining good contact at the alkali metal/SSE interface in practical applications will remain a crucial objective and the introduction of a buffer layer only shifts the problem from Li/SSE to Li/buffer layer wetting. Studying the wettability of protective interlayers adds a level of complexity (both experimentally and computationally) to the initial work, consisting of assessing their thermodynamic stability in contact with Li metal.

**Figure 26:**
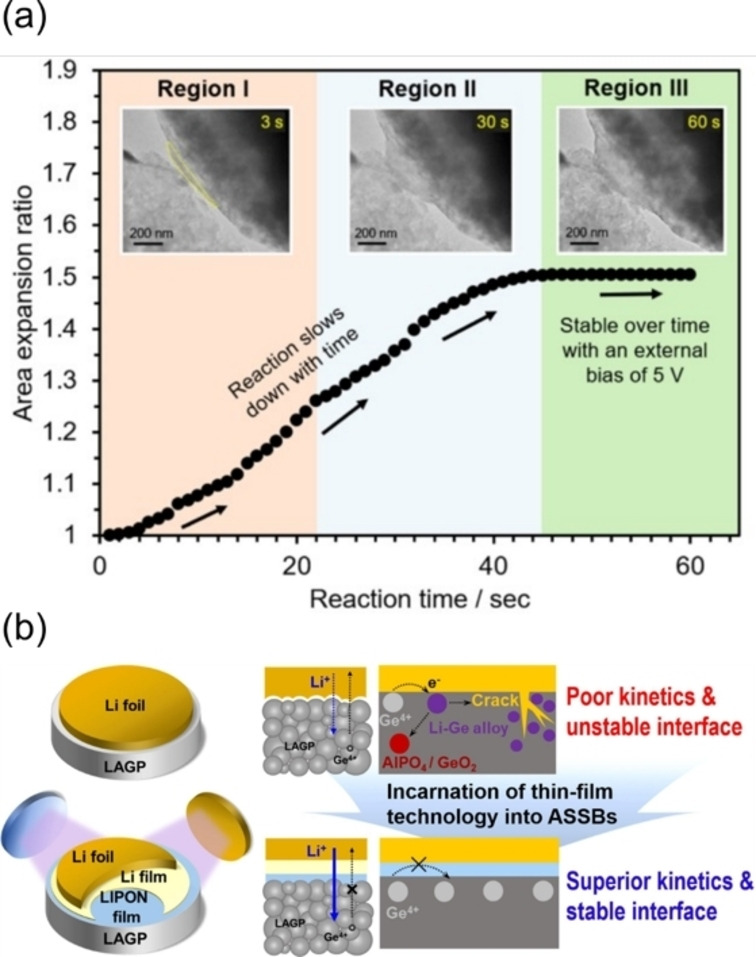
(a) Reactive wetting captured at the Li/LiPON interface via in situ TEM. Reproduced from Ref. [280]. Copyright 2021 American Chemical Society. (b) LiPON can act as a protective interlayer for LAGP and a contact mediator via reactive wetting. Reprinted with permission from Ref. [276]. Copyright 2022 Elsevier.

One candidate material which could provide these combined benefits of SSE protection and Li metal adhesion is LiPON.^[276]^ Although LiPON is not intrinsically stable against Li metal, it decomposes to form a stable SEI which would provide a barrier against the decomposition of more unstable SSEs (such as LAGP; Figure 26). The decomposition reaction at the LiPON/Li interface may therefore serve as a reactive wetting mechanism to improve interfacial contact. A microscopy study by Hood et al. succeeded at capturing the spreading of the SEI at the LiPON/Li interface thanks to an in situ TEM setup. The study demonstrated that the interfacial contact area between Li and LiPON increases in the first minute after contact between the two phases. Although this strategy provides an elegant solution to protect unstable SSEs and improve interfacial contact all at once, it is still unclear whether the SEI forming upon using LiPON as an interlayer can prevent void formation at high current densities. In particular, the presence of Li_2_O in the LiPON SEI indicates that the work of adhesion of Li metal against the SEI‐interlayer is likely to be weak.^[104]^

As discussed in Section 3.1, sulfide electrolytes typically have very small electrochemical stability windows and show rapid decomposition against alkali metals unless protected. In the LGPS system, a lithiophilic‐lithiophobic gradient interlayer was used, formed through the reduction of Mg(TFSI)_2_LiTFSI in DME liquid electrolyte (DME=dimethoxyethane, which is later evaporated). A Li_x_Mg alloy forms on the lithium anode side and a Li‐F‐rich layer sits next to the LGPS, with a polymer in between the two.^[277]^ Tu et al. have in situ formed a dense Li_3_N layer as an interface between the lithium metal anode and an N‐doped Li_6_PS_5_Cl electrolyte, resulting in enhanced cycling stability.^[278]^ The use of Li_3_N as a stable additive to improve the wetting of Li metal in liquid electrolyte systems was previously studied by Park and Goodenough in a conventional liquid electrolyte system.^[279]^

Using a different interlayer chemistry, a group of researchers from Samsung also demonstrated the performance of a Ag‐C nanocomposite buffer layer to protect the Li_6_PS_5_Cl/Li interface.^[244]^ Their study demonstrates that the Ag nanoparticles form an alloy with Li which helps its homogeneous plating at the interface and that the C layer acts as a buffer layer protecting the Li_6_PS_5_Cl electrolyte from reduction. Interestingly, their study also shows that, after a few cycles, the Ag initially contained in the interlayer can be found across the entire thickness of the Li metal electrode, suggesting that alloying interlayers may not be morphologically stable upon repeated cycling.

#### 4.3.5 Challenges and limitations of interlayers

Despite the great popularity of interlayers to improve wetting, very few studies have focused on their long‐term performance over hundreds or thousands of cycles. If reactive wetting is the mechanism by which a good wetting of the interlayer is obtained, then a risk to take into account is the progressive diffusion of the interlayer elements away from the metal/SSE interface into the bulk of the metal electrode. This dissolution of the interlayer into the bulk of the metal anode is strongly suggested by the few studies which looked at the long term stability of reactive interlayers.[^158^, ^240^, ^244^, ^254^, ^281^, ^282^] As the interlayers dissolve into the metal electrode, adhesion between the SSE and metal electrode gradually decreases, which could favor the coalescence of voids during stripping. For this reason, reactive interlayers were qualified as cell assembly “contact mediators” by Krauskopf et al.^[283]^ To mitigate this problem, Jin et al. have suggested to increase the interlayer thickness,^[254]^ while other authors have suggested strategies to minimize dissolution. For instance, Feng et al. found that adding Cu to a Li‐Sn alloy allowed the interlayer to maintain its morphological stability.^[240]^ Another strategy used by Gross et al. to immobilize a Sn interlayer at the interface between Na metal and Na_3_Zr_2_Si_2_PO_12_ consists of operating the battery above the melting point of Na metal^[284]^ in a region of the Na‐Sn phase diagram where the solubility of Sn in Na is minimal.^[285]^

Another important limitation to the commercial deployment of interlayer strategies is the requirement for advanced thin film deposition techniques such as ALD, plasma enhanced chemical vapor deposition (PECVD) or magnetron sputtering.^[286]^ Whilst thin film deposition techniques are widely used within the semiconductor industry, significant scientific and engineering developments, in addition to large commercial investment, would be required for these techniques to be practically used in commercial SSB manufacturing.^[287]^

### 4.4 Alloy anodes

Whilst alkaliphilic interlayers are efficient at facilitating good adhesion, their long‐term stability and dissolution in the metal anode is debated. If the dissolution of alloying interlayers is considered inevitable, then using alkali metal alloys with a sufficiently high concentration of alloying elements as starting anode materials could provide a solution to maintain the interface morphological stability. The alloying of alkali metals with heavier elements not acting as active charge carriers implies that alloy anodes bear an energy density penalty in comparison to pure alkali metal anodes. But this penalty may be balanced by the benefits of maintaining the interface's integrity during cycling.

**Figure 27:**
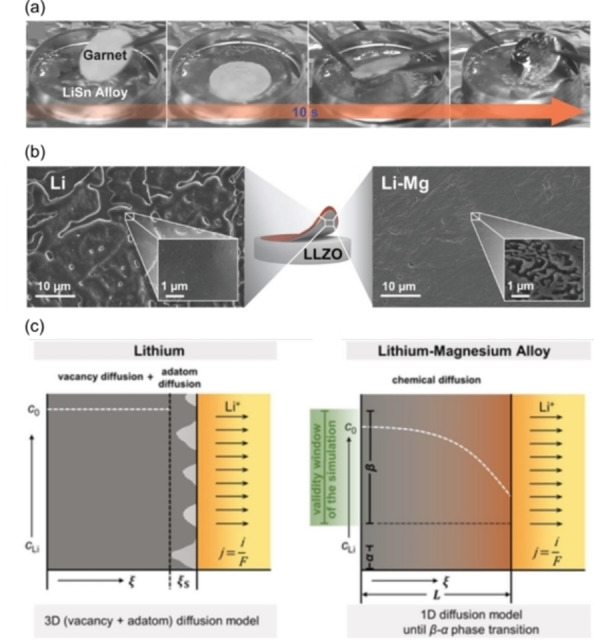
(a) Photographs demonstrating that a LiSn alloy can rapidly spread and coat a LLZO surface. Adapted from Ref. [239]. Copyright 2017 Wiley‐VCH. (b) Surface SEM images of metal electrodes captured after a long stripping experiment and showing the formation of microscopic voids on a pure Li metal surface (left) and that voids are much smaller for the Li‐Mg alloy surface (right). Adapted from Ref. [283]. Copyright 2017 Wiley‐VCH. (c) Schematic illustration of the diffusion models which can be used for a pure Li metal electrode versus a Li‐Mg alloy electrode. The rate performance under stripping becomes limited by Li diffusion through the β phase of the Li‐Mg alloy at the interface (if stripping occurs too fast, an unwanted Li deficient α phase of the alloy will form at the interface). Adapted from Ref. [283]. Copyright 2017 Wiley‐VCH.

On the Li side, Li‐Al,[^288^, ^289^, ^290^] Li‐Mg,^[283]^ Li‐In^[288]^ and Li‐Sn^[239]^ are some of the common Li alloys whose wettability against SSEs has been investigated (Figure 27). Mixing graphite powder in Li metal has also been reported to improve the stability of the Li/LLZO interface.^[291]^ On the Na side, Na‐Cs,^[292]^ Na‐Sn,^[251]^ Na‐Bi,^[251]^ and Na‐In^[251]^ have all been investigated in combination with Na‐β/β’’‐Al_2_O_3_, although only above the melting point of alloys. In all studies, using alloy anodes led to amelioration in contact angles or reduction in interface resistances. As for the cause of wetting improvements, Lu et al. demonstrated using DFT that the superiority of the Na‐Cs alloy in comparison to pure Na metal was associated to its stronger work of adhesion versus Na‐β/β’’‐Al_2_O_3_.^[292]^

An objective for future studies will involve finding an optimum content of alloying element in the metal anode, whilst not degrading the interface stability. Good adhesion is sometimes achieved at the expense of energy density with some reported alloys still employing a marginal fraction of useable charge carriers (1 : 4 atomic fraction for the Na‐Cs alloy,^[292]^ or 30 : 70 weight fraction for best performance of the Li‐graphite paste^[291]^). The effort is already ongoing with studies reporting more industrially relevant alloy compositions such, as the Li_0.9_Mg_0.1_ alloy.^[283]^

Alloys do not always have a principal role of promoting contact at the anode/SSE interface. The lower electrochemical potential of some alloys in comparison to pure metallic Li/Na is exploited to mitigate the reduction of SSEs with narrow stability windows. The efficiency of alloys at protecting sulfide electrolytes (such as LGPS or Li_6_PS_5_Cl) against reduction was demonstrated in several studies.[^288^, ^289^, ^290^]

With respect to wetting, alloys are characterized by their capacity to maintain contact at the alloy/SSE interface. Krauskopf et al. demonstrated that polarization under cycling conditions of a Li‐Mg alloy was delayed in comparison to pure metal, due to a suppression of microvoids.^[283]^ Yet, the Li‐Mg still showed limitations: Li diffusion through the alloy was demonstrated to have become the rate limiting step preventing fast anodic stripping rates (Figure 27).^[283]^ Operating the Li/LLZO battery at higher temperatures (up to 100 °C) was proposed as a solution to reach higher discharge rates.

### 4.5 Interface microstructure engineering

Up until this point in the review, solutions to increase alkali metal/SSE adhesion have focused on strategies involving a modification of surface chemistry. Yet, numerous examples of liquid/solid interfaces inform us that in addition to surface chemistry, surface topography can play a major role in wetting (see Section 2.1.5). Surface topography engineering is a less exploited route to improve the wettability of SSE, which has nonetheless recently proved its efficacy.^[293]^

#### 4.5.1 Impact of SSE roughness

Several studies have demonstrated that the SSE surface roughness has an important impact on the interfacial resistance, but the impact of the surface roughness on the interfacial adhesion in these systems is often not well understood. For the Li/LLZO interface, various studies have now demonstrated that the interface resistance could be minimized by employing roughly polished LLZO pellets.[^235^, ^294^, ^295^] A study from Otto et al. demonstrated the presence of contaminants on the surface of Li metal and suggested that the plastic deformation of the Li metal on the protrusions of a rough SSE surface can break the passivation layer, thus creating unobstructed ion transfer pathways.^[235]^ Quérel et al. have also proposed the same mechanism to explain why the Na/NZSP interface resistance was lower when the NZSP surface had a higher roughness.^[131]^

Contrary to these previous studies, Wang et al. reported for the Li/LAGP interface that an ultrafine polishing of the LAGP pellet is required to optimize wetting, guarantee a uniform plating and suppress dendrites growth.^[296]^ Likewise, Wang et al. demonstrated that the wetting of liquid Li metal on Ni substrates was improved on smooth Ni foils in comparison to porous Ni foams.^[75]^

#### 4.5.2 SSE scaffolds

To achieve high apparent (cell level) current densities while maintaining low local (interface level) current densities, scaffold structures based on porous SSEs whose cavities can be filled by alkali metal have been developed. These structures benefit from an enlarged metal/SSE contact area in comparison to a planar configuration, thereby enabling high apparent current densities without void formation.^[297]^

**Figure 28:**
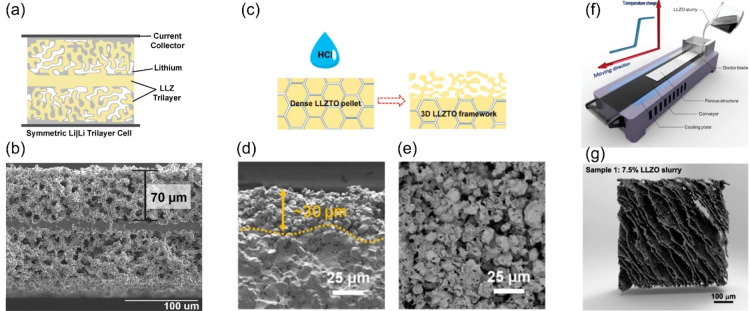
Examples of SSE scaffold structures: (a) schematic depiction and (b) cross‐section SEM image of LLZO trilayer structure produced by tape casting with a sacrificial pore former. Adapted with permission from Ref. [297]. Copyright 2018 Elsevier. (c) Schematic depiction, (d) cross‐section, and (e) top‐view SEM image of a LLZO porous structure produced via acid etching. Adapted from Ref. [299]. Copyright 2020 American Chemical Society. (f) Schematic depiction and (g) 3D reconstruction of a LLZO scaffold produced by freeze tape‐casting. Adapted with permission from Ref. [301]. Copyright 2020 American Chemical Society.

Porous SSE scaffolds can be obtained by mixing sacrificial pore formers with the SSE before sintering; the decomposition of the pore former during sintering creates a random percolating network of pores (Figure 28).[^248^, ^297^, ^298^] HCl acid etching has also been reported to produce a network of pores on the surface of LLZO (Figure 28).^[299]^ More ordered structures can be obtained by 3D printing the scaffold on top a dense SSE layer.^[300]^ One of the best demonstrations of the efficiency of such anode scaffolds for fast charging applications was provided by Hitz et al. who were able to reversibly cycle a Li/LLZO/Li symmetrical cell at a current density of 10 mA cm^−2^ and a plating capacity of 1.67 mAh cm^−2^ without short‐circuits or signs of large polarization.^[297]^ These porous structures do not improve the intrinsic wetting properties of the SSE, and alkaliphilic interlayers (such as Al_2_O_3_ ^[297]^ or ZnO[^248^, ^298^, ^299^]) were employed in all the previously reported studies to improve the wetting of the metal inside the pores of the SSE scaffold. Thus, these structures only redistribute the formation of voids across a larger interface area.

Compared to pure alkali metal electrodes, the drawback of composite metal/SSE electrodes is their reduced energy density. Future studies are required to assess whether SSE scaffolds with a higher pore volume fraction can be produced and if the resulting metal/SSE composites have the same mechanical integrity and electrochemical performance. For example, Shen et al. have recently employed freeze‐tape‐casting as a technique to produce 75 % porous LLZO scaffolds (Figure 28).^[301]^

#### 4.5.3 Non‐SSE scaffolds

Besides SSE scaffolds, other materials have been considered for hosting the plating and stripping of alkali metals. These materials can be either electronic conductors or mixed ionic‐electronic conductors (MIECs). The largest subsection of publications referring to scaffold structures for alkali metal anodes can be found in the field of liquid electrolyte batteries. For liquid electrolyte cells, host structures are being considered for alkali metal electrodes because they could provide a better directional control over Li/Na plating and thus prevent dendrite growth.^[302]^ But these types of host structures are also being investigated for SSBs, as illustrated by the following examples.

**Figure 29:**
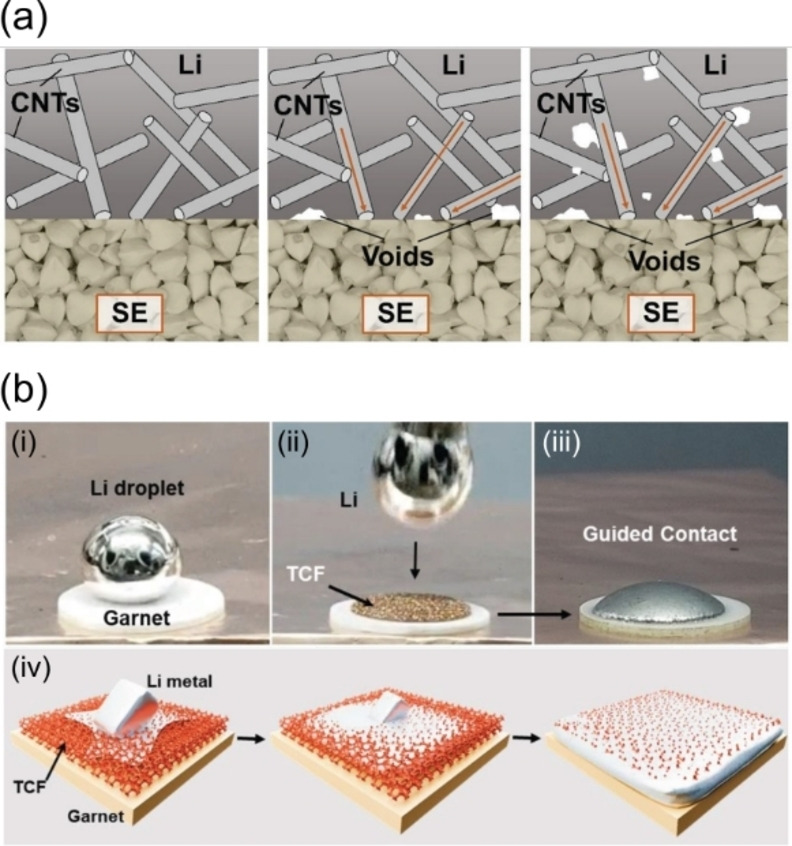
(a) Carbon nanotubes (CNTs) were mixed with Li metal to create a composite structure and delocalize voids away from the Li/SSE interface. Reproduced with permission from Ref. [303]. Copyright 2022 the Authors, published by Wiley VCH. (b) A treated copper foam (TCF) is employed as a host structure to improve wetting Li metal and LLZO: (i) photo of a Li droplet on a bare LLZO surface showing poor wetting; (ii) and (iii) Li metal wets the TCF well and infiltrates the structure as schematically illustrated in (iv). Adapted from Ref. [293]. Copyright 2020 Wiley‐VCH.

In a recent study, Fuchs et al. produced a composite Li/C electrode by mixing C nanotubes in Li metal (Figure 29).^[303]^ They observed that the composite structure did not prevent the formation of voids at the interface, but found that the capacity they could extract from electrodes was greatly improved. They suggest that the carbon nanotubes act as rails guiding Li towards the redox active interface. Although their study highlights the limited performance of the composite structure, the idea of delocalizing the stripping away from the interface and into the bulk of the composite electrode could be a promising strategy for future host structures.

To improve the kinetics of interfacial void annihilation, a slightly different approach was adopted by Chen et al. who did not randomly mix the C nanotubes in the Li metal but looked at the possibility to plate and strip Li metal inside aligned C nanotubes.^[304]^ The inside surface of their C nanotubes was functionalized with a thin ZnO_x_ layer deposited by ALD to facilitate the initial plating and stripping inside the tubules. By looking at the moving front of Li metal inside the nanotubes via an in situ TEM setup, they observed spreading along the walls of the nanotubes as a result of reactive wetting between Li and ZnO_x_. Upon stripping, they noticed that voids could form at the junction between their PEO polymer electrolyte and C nanotube, but that this did not prevent stripping completely. They suggested that the Li metal diffusion inside the tubules occurred mostly via a Coble creep mechanism (a form of creep preferentially occurring along interfaces and grain boundaries).^[304]^

Duan et al. investigated another host structure chemistry consisting of a copper foam which was thermally treated in air to form a lithophilic CuO_x_ surface.^[293]^ They demonstrated that their host structure could be used to constrain alkali metals to maintain good contact with a LLZO electrolyte (Figure 29). They also demonstrated that the denser the pattern, the greater the adhesion between the composite electrode and LLZO. The benefits of the copper foam are confirmed by the large reduction of interface resistance in comparison with an untreated surface (from 998 to 9.8 Ω cm^2^) and by its stable cycling performance at 0.3 mA cm^−2^ for more than 600 h.

Engineering host structures with fast diffusion along their walls could be a solution to facilitate the access of bulk Li or Na metal to the redox active interface. Theoretical studies should investigate this field to identify the ideal properties of the metal/host composite (e. g., metal/host interface energy, host structure, pore size, …) and guide the discovery of new host structures.

## 5 Conclusions and Future Outlook

This Review provides a fundamental understanding of the factors that govern interfacial adhesion or ‘wettability’ at solid/solid interfaces in solid‐state batteries (SSBs). Particular attention is given to the alkali metal/solid‐state electrolyte interface, which is a crucial component of next‐generation SSBs with high energy and power densities. The suppression of alkali metal void formation at the alkali metal/SSE interface during electrochemical stripping at industrially relevant current densities has been a key challenge for the commercial realization of SSBs. The application of high pressures to suppress void formation still poses significant engineering challenges. As we highlight throughout this review, careful control and understanding of the interfacial chemistry and adhesion may offer a step forward towards the practical adoption of SSBs with alkali metal anodes, without the requirement for high pressures.

Interfacial adhesion, or ‘wetting’ at metal/ceramic and metal/metal interfaces is a thermodynamically controlled property that has been studied for over half a century in a diverse range of material science applications from corrosion to thermal barrier coatings and biomedical applications, providing a wealth of previous knowledge that can be translated to SSB chemistries. The Gibbs free energy of the reaction between participating phases will determine if the wetting between two materials is reactive (ΔG <0) or nonreactive (ΔG >0). As we have demonstrated throughout this review, the distinction between these regimes has important implications for the time evolution and chemical composition of the interface. The strongly reducing potential of Li and Na metal renders the interfaces with the majority of common SSE materials ‘reactive’, leading to SSE decomposition and the formation of secondary phases (Li_2_O, Li_2_S, Na_3_P, etc). Good interfacial adhesion must also be maintained at the newly formed ‘nonreactive’ interface between the alkali metal and the secondary phases.

Contact angle (CA) measurements are ubiquitous in the surface science field for understanding the interaction between a liquid and a substrate, in which the interfacial work of adhesion (*W*_ad_) can be related to the contact angle (θ ), and liquid surface energy, $\sigma_{LV}$ , through the Young–Dupré equation, *W*_ad_=$\sigma_{LV}$ (1+cosθ ). It has become commonplace in the experimental literature to use the approximate contact angle of molten droplet of Li or Na on a SSE as an indication of the interfacial adhesion. However, care must be taken with this approach as Young–Dupré equation is only applicable for perfectly flat, nonreactive interfaces, a condition which is rarely met in SSE systems. The development of more sophisticated CA measurements for alkali metal systems under controlled atmospheres are required to provide quantitative values for the work adhesion with well‐defined surfaces of SSE materials.

The continuous development of computational methods and computing power over the last decade has provided invaluable tools for understanding the structure and reactivity of interfaces. The Gibbs free energy of reactions between bulk alkali metals, SSEs and interlayer phases can be readily calculated with density functional theory (DFT) to either design systems with nonreactive interfaces, or deliberately engineer in reactive interfaces with increased wetting. The calculation of explicit surfaces and interfaces with atomistic calculations can give detailed insights into the changes in electronic structure and binding at interfaces, in addition to changes in the local structure because of atomistic mismatch between solid phases. Values for the work of adhesion/separation between selected surfaces of alkali metals and SSE can readily be calculated with DFT calculations, although future efforts should be directed towards validating computational values with careful experimental contact angle measurements on well‐defined model interfaces.

The inherently dynamical nature of alkali metal/SSE interfaces can be captured through state‐of‐the‐art molecular dynamics and/or transition state theory simulations. However, challenges remain with the system sizes that can be studied due to the significant computational expense of explicit interface calculations containing 100s of atoms with DFT. The large computational expense typically means that only a limited number of high symmetry alkali metal/SSE interface orientations have been investigated, even for the most well studied SSE systems. Future work should focus on the development of more realistic, larger scale models to understand the nature of more complex interfaces containing extended defects, such as dislocations. The further development of structure prediction methods and machine learning models coupled with density functional theory calculations for the prediction of interfacial structures and inexpensive evaluation of the energies and dynamics is a highly promising avenue to tackle this problem.

At the particle level, phase field simulations have proven to be an invaluable tool for studying the evolution of the morphology alkali metal anodes. Only a handful of studies have investigated the specific role of interfacial adhesion on model alkali metal/SSE interfaces. Future studies should focus on the development of phase field models studying more complex effects such as the role of SSE surface morphology and roughness on alkali metal adhesion in addition to aiding the design of novel 3D scaffolds.

The work of adhesion between solid alkali metals and SSEs is intrinsically linked to the formation energy of alkali metal vacancies at the alkali metal/SSE interface. Weak interfacial adhesion and disorder leads to the accumulation of alkali metal vacancies at the interface which can coalesce into voids, whereas strong interfacial adhesion results in the injection of vacancies away from the interface, promoting interfacial contact. First principles calculations indicate that contact angles approaching θ= 0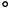 , (*W*_ad_ $\geq 2\sigma_{Li}$ ) i. e. perfect wetting are required to suppress vacancy coalescence at the interface.

The work of adhesion between alkali metals and SSEs, or other components such as interlayers or current collectors, depends strongly on the nature of bonding at the (nonreactive) interface. The bonding at the interface between metals and ionic ceramics is intrinsically weak as it is primarily governed by weak electrostatic interactions. An important consequence of this is that none of the state‐of‐the‐art ceramic SSE materials such as Li_7_La_3_Zr_2_O_12_, Li_6_PS_5_Cl, Na‐β’’‐Al_2_O_3_ or common decomposition products such as Li_2_O, Na_2_O, Li_3_PO_4_ and Li_3_P and LiCl meet the criteria of θ= 0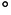 . Direct modification of the SSE chemistry to improve the interfacial adhesion is, however, challenging. Increasing the covalency of the bonding within the SSE material will typically lead to enhanced wettability with alkali metals, but also typically increases the reactivity (i. e. ΔG <0) leading to decomposition. The introduction of open d‐shell cations such as Zr^4+^ is a more promising strategy to improve the interfacial adhesion while maintaining a ‘nonreactive’ system, but as can be seen from systems such as Li_7_La_3_Zr_2_O_12_, the criteria of θ= 0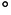 is still not fully met.

The presence of ionic or ionocovalent contaminants such as Li_2_CO_3_, LiOH and Li_2_O on the surface of SSE materials, have been shown to further reduce the work of adhesion compared to pristine SSE systems. A fundamental understanding of the surface chemistry of SSE materials is therefore essential, requiring the development advanced surface characterization tools that can probe the chemistry and morphology of surface phases, both before and after electrochemical cycling. The reactivity of many SSE materials in ambient environments means that careful control of atmospheric conditions and sample handling is essential for reproducible measurements of interfacial properties.

A wide variety of strategies to improve the alkali metal adhesion have been explored in the literature, but they can broadly be categorized into: interlayers, alloy anodes, scaffolds and morphological control (i. e. roughness). The introduction of a thin interlayer material between the alkali metal and SSE that has a strong interfacial adhesion with the alkali metal has been studied extensively by many authors, although the mechanism by which the interlayers improve the performance is sensitive to the interlayer chemistry. In contrast to metal/ceramic interfaces, the intrinsically strong nature of metallic bonding and high surface energies of metals and alloys leads to strong adhesion at metal/metal interfaces. The introduction of metallic alloy interlayers has therefore been a particularly successful approach. However, dissolution of the interlayer into the bulk metal can cause serious limitations to the long‐term stability of metallic interlayers, limiting the performance of such strategies. Ensuring that the interlayer remains located at the interface is an important consideration for future materials design strategies.

The majority of metallic interlayers employed to improve contact of metal/SSE interfaces act via a reactive wetting mechanism. Whilst reactive wetting can be extremely efficient to improve contact in the first hours after cell assembly, it is important to also consider the stability of such interlayers in the long‐term. More specifically, if an interlayer is employed to prevent void coalescence, then it should remain fixed at the metal/SSE interface for hundreds or even thousands of cycles without dissolving into the metallic electrode. The adhesion of the interlayer to the SSE also needs to be carefully controlled to avoid a delamination during metal plating.

Polymer interlayers are also starting to being investigated on the basis that their good deformability could be an advantage to accommodate the large volume changes of the metallic electrode. A lot of research is still required in this area to control wetting fundamentally and experimentally at the polymer/metal and polymer/SSE interfaces.

Beyond planar interfaces, a lot of studies are now starting to investigate composite negative electrodes. This includes strategies where the alkali metal is plated in the pores of a SSE scaffold. By delocalizing the stripping current density to a larger contact area, current constriction and the risk of metal filament nucleation can be efficiently mitigated. Yet, composite structures with a non‐negligible volume fraction of dense SSE significantly reduce the energy density of cells. Ionically inert host structures are also being investigated and fast diffusion of Li/Na along the host/metal interface could be a future strategy to delocalize stripping away from the planar redox active interface into the bulk of the metallic electrode.

For extremely high‐power applications, a solution becoming more and more promising is to melt the metallic anode, as void annihilation has been demonstrated to be extremely efficient in SSBs operating above the alkali metal melting point. Wetting mediators or host structures are, however, often still required even for high temperature SSBs.

Finally, the study of interfacial adhesion has a rich history that spans many disciplines across the field of material science and chemistry. Further advances in our fundamental understanding and the development of both experimental and computational tools to tackle interfacial adhesion in the context of solid‐state batteries will reduce the time to commercialization of these devices, but will also have wider applications to the material science community from corrosion science to nuclear energy and biomaterials. We therefore strongly encourage broader collaborations between battery scientists and scientists and engineers outside the battery community to develop novel solutions to interfacial adhesion challenges that will help to facilitate the adoption of sustainable battery technology that will benefit all of society.

## Conflict of interest

There are no conflicts of interest to declare.

## Biographical Information

*Dr. Ieuan D. Seymour is a Lecturer at the University of Aberdeen. He was previously a postdoctoral researcher at Imperial College London and the University of Texas at Austin after completion of his Ph.D. in Chemistry at the University of Cambridge. His research focuses on understanding the structural properties of commercial and next‐generation electrode and electrolyte materials for rechargeable batteries and solid oxide fuel cells. He uses a combination of experimental techniques, including solid‐state nuclear magnetic resonance, and first‐principles calculations to understand the role of local structure on the electrochemical performance of novel materials for renewable energy applications*.

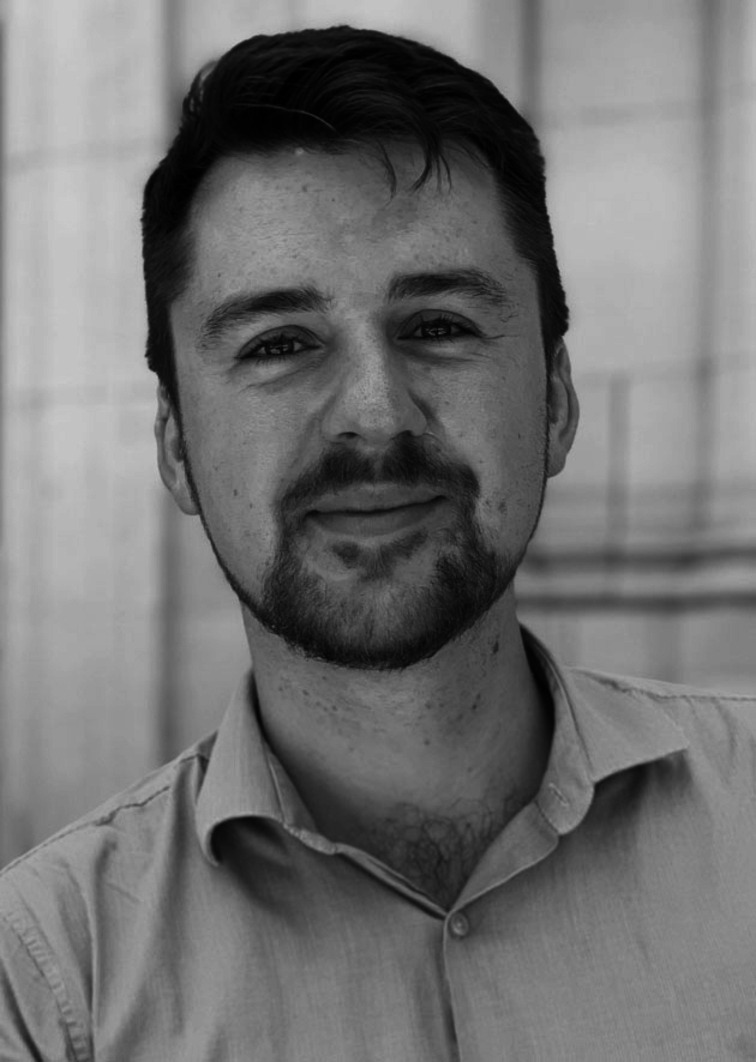

## Biographical Information

*Dr. Ainara Aguadero is a Tenured Scientist at the Instituto de Ciencia de Materiales de Madrid at the Consejo Superior de Investigaciones Cientificas in Spain ICMM‐CSIC and Visiting Reader in Energy Materials in the Department of Materials, Imperial College London. Her research focuses on the quantitative analysis and optimization of ion and electron dynamics in complex oxides, bulk, surfaces, and interfaces. She uses a combination of structural, chemical and electrochemical analysis including surface‐sensitive techniques and operando characterization to develop the next generation of solid‐state electrochemical devices, such as all‐solid‐state batteries and intermediate temperature solid oxide fuel cells and electrolyzers*.